# Exploring Metal–Organic Framework-Coated Blades for Direct and High-Throughput Screening Analysis of Complex Biological Matrices

**DOI:** 10.1021/acs.analchem.5c02782

**Published:** 2025-08-20

**Authors:** Isaac Negrín-Santamaría, María J. Trujillo-Rodríguez, Encarnación Moyano, Juan H. Ayala, Olatz Zuloaga, Jorge Pasán, Verónica Pino, Juan F. Ayala-Cabrera

**Affiliations:** † Laboratorio de Materiales para Análisis Químico (MAT4LL), Departamento de Química, Unidad Departamental de Química Analítica, 16749 Universidad de La Laguna (ULL), San Cristóbal de La Laguna 38108, Spain; ‡ Unidad de Investigación de Bioanalítica y Medioambiente, Instituto Universitario de Enfermedades Tropicales y Salud Pública de Canarias (IUETSPC), ULL, San Cristóbal de La Laguna 38108, Spain; § Department of Chemical Engineering and Analytical Chemistry, University of Barcelona (UB), Barcelona 08028, Spain; ∥ Department of Analytical Chemistry, University of the Basque Country (UPV/EHU), Leioa 48940, Spain; ⊥ Research Centre for Experimental Marine Biology and Biotechnology (PiE-UPV/EHU), University of the Basque Country, Plentzia 48620, Spain; # Laboratorio de Materiales para Análisis Químico (MAT4LL), Departamento de Química, Unidad Departamental de Química Inorgánica, Universidad de La Laguna (ULL), San Cristóbal de La Laguna 38108, Spain; ∇ Centro de Investigación en Red de Enfermedades Infecciosas (Ciberinfec), Instituto de Salud Carlos III, Madrid 28049, Spain

## Abstract

Ambient ionization mass spectrometry (AIMS) techniques have become an emerging approach over the last years due to their simplicity, permitting high-throughput sample analysis by directly coupling with mass spectrometry while ensuring short analysis times. Among AIMS techniques, coated-blade spray (CBS) has stood out, as it ensures a significant enhancement of overall sensitivity, undoubtedly useful for human biofluid analysis. In parallel, the incorporation of advanced smart materials, such as metal–organic frameworks (MOFs), into analytical devices is increasing due to their outstanding ability to efficiently trap target analytes, such as industrial chemicals with diverse functionalities and polarities. This study integrates neat MOFs in CBS devices, without the need for any composite or additional materials, through a simple and mild strategy and shows their use in the determination of xenobiotics present in human urine samples. Moreover, a suspect screening workflow by high-resolution mass spectrometry (HRMS) has been developed for the first time to extend the chemical coverage of AIMS techniques. This simultaneous approach ensures a proper analytical quality performance, achieving limits of quantification (LOQs) down to 0.1 ng·mL^–1^ despite requiring only 8 min for the entire procedure.

## Graphical abstract

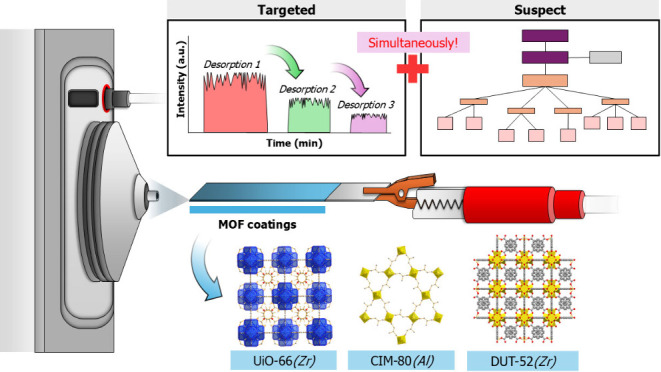

## Introduction

Since its development in 2004, ambient ionization mass spectrometry (AIMS) has become a very attractive approach for the direct analysis of samples without the requirement of a chromatographic separation. AIMS techniques ensure impressive laboratory workloads due to the high sample throughput in real time and the simplicity, usually achieving complete analysis in less than a minute in an open mass spectrometry system.^1,2^ In particular, AIMS has become an important tool for applications such as food analysis^1^ and drug monitoring.^3–5^

The main drawback of AIMS techniques is the sensitivity, presenting relatively high limits of quantification (LOQs) in the range of mg·L^–1^. This is particularly problematic when dealing with complex matrices such as biofluids,^3^ especially if considering that human biofluids currently constitute the most obvious link between the exposure to chemicals (chemical exposome) and the metabolic changes in an organism (metabolome).^6,7^ Indeed, the monitoring of endogenous and exogenous compounds (i.e., xenobiotics) is a common strategy for establishing those relationships.^8^ The concentration of xenobiotics in human biofluids is normally much lower than the concentration of endogenous substances, which may lead to analytical issues when using AIMS techniques. Moreover, the chemical exposome and metabolome comprise a vast number of substances, requiring the use of suspect screening approaches to expand the knowledge on this field.^9^ Thus, there is a need to broaden the view of AIMS analysis to make it compatible with the determination of low-abundance xenobiotics and wide-scope analysis of endogenous and exogenous substances in human biofluids.

In recent years, the inclusion of a fast extraction step before AIMS analysis is gaining more interest to bypass some of these disadvantages. These approaches, also known as microextraction-AIMS (μe-AIMS), aid to remove interferences that can affect the ionization efficiency, while they comprise preconcentration of the analytes, ultimately improving the sensitivity.^3^ μe-AIMS approaches can be classified according to the type of substrate, interface, and desorption/ionization mechanism.^3,4,10,11^ Among them, coated-blade spray (CBS) techniques stand out as one of the most promising μe-AIMS approaches due to their simplicity, ease of automation, and even applicability for on-site analysis with portable MS systems.^2^ CBS could be considered as an integrated μe-AIMS since it allows both the extraction and the analysis on the same substrate and, thus, improves the throughput. CBS devices consist of a solid support, commonly a metallic blade coated with a sorbent material.^12,13^ This blade is initially exposed to a sample for the extraction of the analytes, consecutively rinsed, and placed in front of the MS inlet for the desorption/ionization step. The use of a blade as support provides CBS with some significant advantages over other μe-AIMS techniques, such as paper spray (PS), like the generation of a more stable electrospray because of the high conductivity, superior mechanical stability, the possibility to rinse the extracted sample to further remove coisolated interferences, or even its direct use as sampling probes for the analysis of tissues, among many others.^14,15^

CBS devices are usually coated with a sorptive composite formed by a particulate sorbent in a polyacrylonitrile matrix.^15–20^ Although these coatings have proven validity for target analysis, it would be ideal to have a less discriminative sorbent when also dealing with suspect screening to expand the chemical space.^20^ Thus, the evaluation of novel materials such as metal–organic frameworks (MOFs) could help to open the door for high-throughput suspect screening in human biofluids.^21^ MOFs are crystalline porous materials composed of metallic centers (either ions or clusters) linked by organic ligands through coordination bonds, defining frameworks with permanent porosity. Their well-defined structure, outstanding surface areas, almost-infinite number of combination of metals and ligands, and chemical and thermal stability make them perfect candidates for use in analytical devices.^22,23^ The evaluation of MOFs as coatings for the preconcentration step in analytical devices requires the growth of a neat layer of MOF on the support to ensure that the MOF is the only material exposed to the sample. This step is crucial in the production of new neat MOF-based devices, but the powder nature of synthesized MOFs precludes easy processing into devices.^24^ Indeed, in the case of CBS metallic blades, there is only one device reported with a MOF material as part of the coating, but it is just a component in a composite, together with PAN and MXene.^25^ To sum up, its applicability as a CBS coating was only shown for water samples.^25^ In this sense, there is no neat MOF coating for CBS, and clearly, MOF-based CBS must be valid for complex biological samples, as this is a key point to improve. There are also studies involving MOFs for paper spray MS, with paper or glass filters as the support, requiring starch as glue (or blender) to ensure the MOF attachment and uniform distribution in the coating.^26,27^ The inclusion of additional materials as composites is also problematic in terms of possible clogging of the pores of the MOF and unwanted signals coming from the polymer (or its additives) in high-resolution mass spectrometry (HRMS).

This study presents the preparation of neat MOF coatings for CBS, taking advantage of the thiol chemistry, specifically with mercaptoacetic acid (MAA). Among the immense variety of possible MOFs as coatings, their selection for the blades followed the criteria of (1) proper water stability, (2) low toxicity, and (3) a greener synthetic approach. This way, CIM-80­(*Al*),^28^ UiO-66­(*Zr*),^29^ and DUT-52­(*Zr*)^30^ were included in this study.

Besides, MOF-coated blades were tested with complex samples such as human urine for the determination of challenging target xenobiotics such as polar industrial pollutants and nonsteroidal anti-inflammatory drugs (NSAIDs), as well as for the suspect screening of endogenous and exogenous substances. An automated target/suspect analysis workflow has also been developed in this study to fully understand the strengths of the MOF-coated blades, achieving a full characterization of real samples in triplicate and in less than 10 min. To the best of our knowledge, this is the first time that neat MOF-coated blades have been used, not only for target but also suspect screening by CBS-HRMS in human biofluids.

## Experimental Section

### Chemicals and Reagents

Main chemicals are listed in the Supporting Information (Experimental S1). Analytes selected for this study include caffeine (CAF), acetaminophen (ACE), cotinine (COT), and naproxen (NAP) as representative examples of industrial and pharmaceutical products; all were obtained from Sigma-Aldrich (purity < 99.9%). Individual stock solutions were prepared in MeOH, except for CAF, which was prepared in MeOH:H_2_O (1:1, *v*/*v*), all at concentrations of 1000 mg·L^–1^. A mixed solution of all 4 analytes was prepared in MeOH:H_2_O (1:1, *v*/*v*) at 100 mg·L^–1^ and used to prepare intermediate solutions from 10 mg·L^–1^ down to 1 μg·L^–1^. All these solutions were stored protected from light at 4 °C in 2 mL amber vials supplied by Agilent Technologies (California, USA). Daily working standard solutions were prepared by appropriate dilution in ultrapure water or synthetic urine. The deuterated analogues selected were ^2^H_8_-carbamazepine (*d*_8_-CAR), ^2^H_6_-diuron (*d*_6_-DIU), and ^2^H_3_-ketoprofen (*d*_3_-KET), acquired from Sigma-Aldrich (purity < 99.9%). Individual standards were prepared at concentrations of 1000 mg·L^–1^, and from them, two mix solutions were prepared at 500 and 1000 μg·L^–1^, respectively.

### MOF-Coated Blade Preparation

Blades were prepared with pieces of stainless steel (5 × 40 mm^2^) with a thickness of 0.5 mm. The neat MOF coating of the blade surface is achieved by following a series of four main steps. The first step is proper surface cleaning. Blades were thoroughly rinsed with soapy water, deionized water, and ethanol in this order. Then, they were dried in an oven and heated at 550 °C for 3 h. Once at room temperature, the blades were chemically etched by being immersed in a 1 M NaOH solution at 60 °C for 5 min, followed by immersion in a HNO_3_/H_2_SO_4_ (1:3, *v*/*v*) solution for 5 min to ensure a rough surface.^31^ The second step is surface functionalization. The cleaned blades were then functionalized by immersing them in an aqueous solution of 0.58 mM MAA at room temperature for 24 h. Afterward, they were washed with deionized water and ethanol and air-dried. The third step is MOF growth. The blades were then immersed in different solvothermal reactors, with the composition of the solutions in the reactors depending on the specific MOF to be grown (as the precursors and solvents for each MOF are different). Then, reactors were placed into autoclaves to perform the *in situ* growth of the crystalline material on the surface of the blade. The heating temperatures used were also dependent on the type of MOF (Table S1 includes data for the synthesis of each MOF). The last step is blade activation. MOF-coated blades were rinsed with their respective reaction solvent (MOF-dependent), followed by abundant rinsing with ethanol and final activation at reduced pressure in an oven at 120 °C overnight.

### Urine Samples

Urine samples were collected in the early morning from healthy male and female volunteers with a consumption of a rational amount of coffee or caffeine-based drinks (from none to 2–3 cups per day) and the consumption of standard pharmaceutical products. Samples were frozen at −32 °C until use. Informed consent and a validated survey were obtained from each volunteer, and the samples were handled in accordance with the indications of the Ethics Commission for Research and Teaching of the University of the Basque Country (CEISH-UPV/EHU, BOPV 32, 17/2/2014 M10 2022 325 and CEIAB-UPV/EHU, BOPV 32, 14/2/14, M30 2022 326). For the analysis, 300 μL of urine was diluted 1:5 (*v*/*v*) with LC-MS water in 2 mL vials acquired form Agilent Technologies (Santa Clara, California, USA). Besides, a mix of surrogates was added at 500 μg·L^–1^ for a final volume of 1.5 mL. The MOF-coated blade was immersed in the sample, and the extraction (3 min) was performed by applying slight agitation by a shaker, acquired from Edmund Bühler GmbH (Bodelshausen, Germany). Then, the blade was rinsed with ultrapure water for 30 s with a shaker agitator to remove possible organic residues of the coating surface. After that, the blade was dried under a gentle N_2_ stream, and the desorption/ionization of the analytes was achieved under optimum conditions using 50 μL of methanol with 0.1% formic acid at 4 kV for the CIM-80­(*Al*)-coated blade and 40 μL of that solvent at 4 kV for the UiO-66­(*Zr*)- and DUT-52­(*Zr*)-coated blades. After each CBS measurement, MOF-coated blades were cleaned by immersion in 15 mL of MeOH for 15 min with agitation. Additionally, prior to every analysis, blade-blanks were performed to ensure no carryover effect was noticeable.

### Instrumentation

CBS experiments with MOF-coated blades were performed by coupling a lab-made setup, schematically represented in Figure 1, with a Q Exactive Focus Orbitrap (Q-Orbitrap) mass analyzer acquired from Thermo Fisher Scientific (Waltham, Massachusetts, USA), operating under ambient open-air conditions.

**Figure 1:**
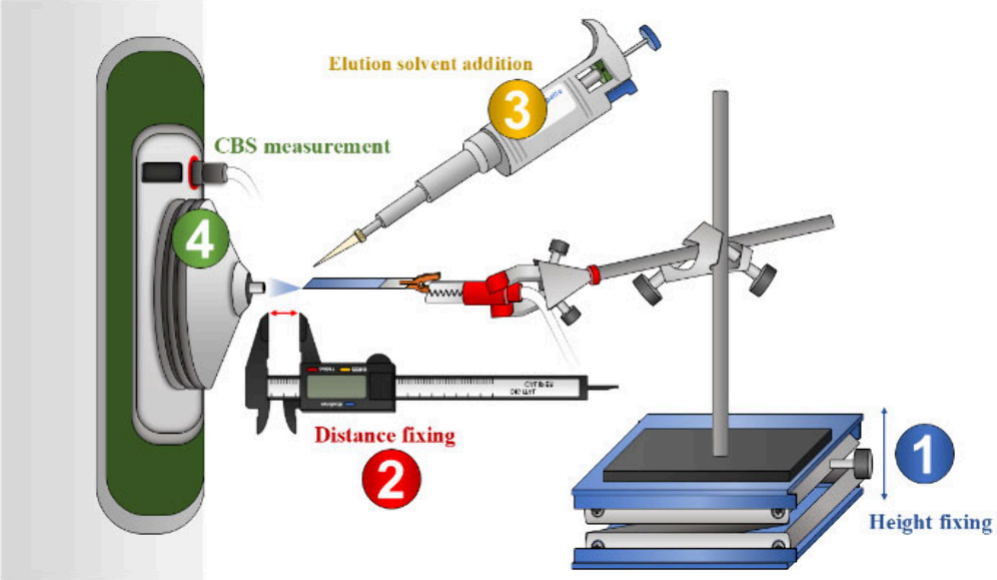
Main setup for the MOF-based CBS experiments.

For both targeted and suspect screening approaches, data were acquired in full scan-data-dependent MS/HRMS acquisition (Full MS-ddMS2) and positive ion mode. For the full scan, the mass range ranged from 120 to 1050 *m*/*z*, and the resolution was set at 70,000 fwhm (at 200 *m*/*z*). For the ddMS2, data were acquired in discovery mode with a resolution of 17,000 fwhm (at 200 *m*/*z*). The quadrupole isolation window was set at 3 *m*/*z*, and the product ion mass spectra were obtained using stepped normalized collision energies at 10, 30 and 70%. The suspect screening workflow is detailed in Procedure S1. Mass calibration was performed daily using the LTQ Velos ESI positive ion calibration solution provided by Thermo using a heated electrospray source (HESI) to ensure mass accuracy. Data acquisition was performed with Xcalibur 4.1 software, while data processing was performed with FreeStyle 1.8 for target analysis and Compound Discoverer 3.3 for suspect screening.

Powder X-ray diffraction (PXRD) data were obtained with an Empyrean PANalytical diffractometer (Almelo, Netherlands) working with Cu Kα radiation (λ = 1.5418 Å) and Bragg–Brentano geometry. Diffraction patterns were acquired at room temperature from 5.00° to 80.00° (in 0.02° steps) with a total exposure time of 12 min. Scanning electron microscopy (SEM) and energy dispersive X-ray spectroscopy (EDX) were performed with an EVO 15 ZEISS SEM (Oberkochen, Germany). All blades were dried and coated with gold to make their surface conductive before performing the analysis. The micrographs obtained were taken at different magnifications (600–1500×), using 7–10 mm as the working distance, 20 kV as the accelerating voltage, and DISS as the digital image recording. FTIR experiments were carried out with an Agilent Cary 630 instrument equipped with an ATR in the spectral range from 650 to 4000 cm^–1^, with 128 scans per sample.

## Results and Discussion

### Device Characterization

The synthesized blades were characterized by PXRD to analyze the crystalline phase on the support, together with the synthesized powder, and compared with the pattern simulated from the crystal structure deposited on the CCDC.^32^ Results confirmed the correct formation of CIM-80­(*Al*), UiO-66­(*Zr*), and DUT-52­(*Zr*) on the blades, as shown in Figure 2. The entire discussion of the obtained PXRD profiles is included in Procedure S2. Besides, IR studies have been also used to complement the characterization (Figure S1), obtaining the same profiles for MOFs and MOF-coated blades.

**Figure 2:**
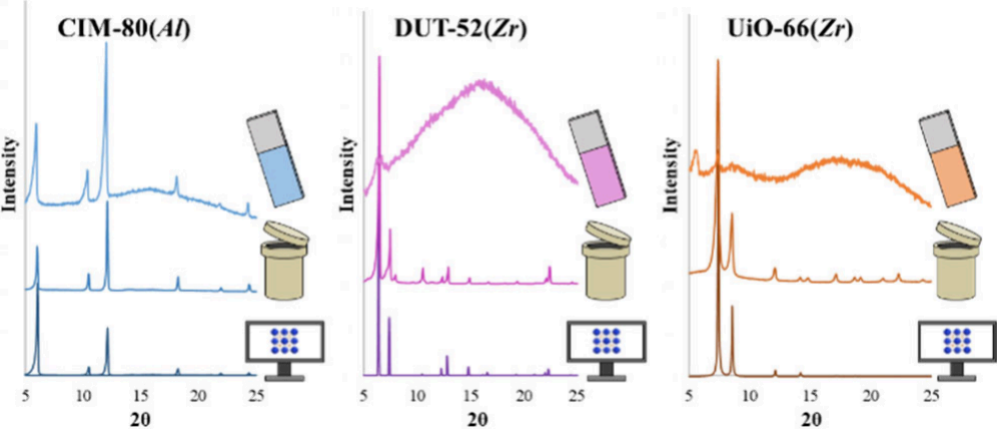
PXRD characterization of the MOF-coated blades.

Surface characterization was performed by scanning electron microscopy (SEM) to provide conclusions about the performance of different surface treatments and chemical etchings and coating homogeneity with the different MOFs. Three different cleanup strategies were evaluated to ensure adequate support for further MOF growth: (1) HNO_3_ at room temperature for 5 min, (2) H_2_O_2_ at 70 °C for 2 h,^22^ and (3) a mixed method with NaOH at 60 °C for 5 min, followed by a HNO_3_/H_2_SO_4_ (1:3, *v*/*v*) washing for 5 min.^31^ Besides, a nontreated blade was also studied for comparative purposes. The best results were obtained with the most aggressive strategy (3), which was able to generate homogeneous surface roughness (Figure 3A, with more details in Figure S2).

**Figure 3:**
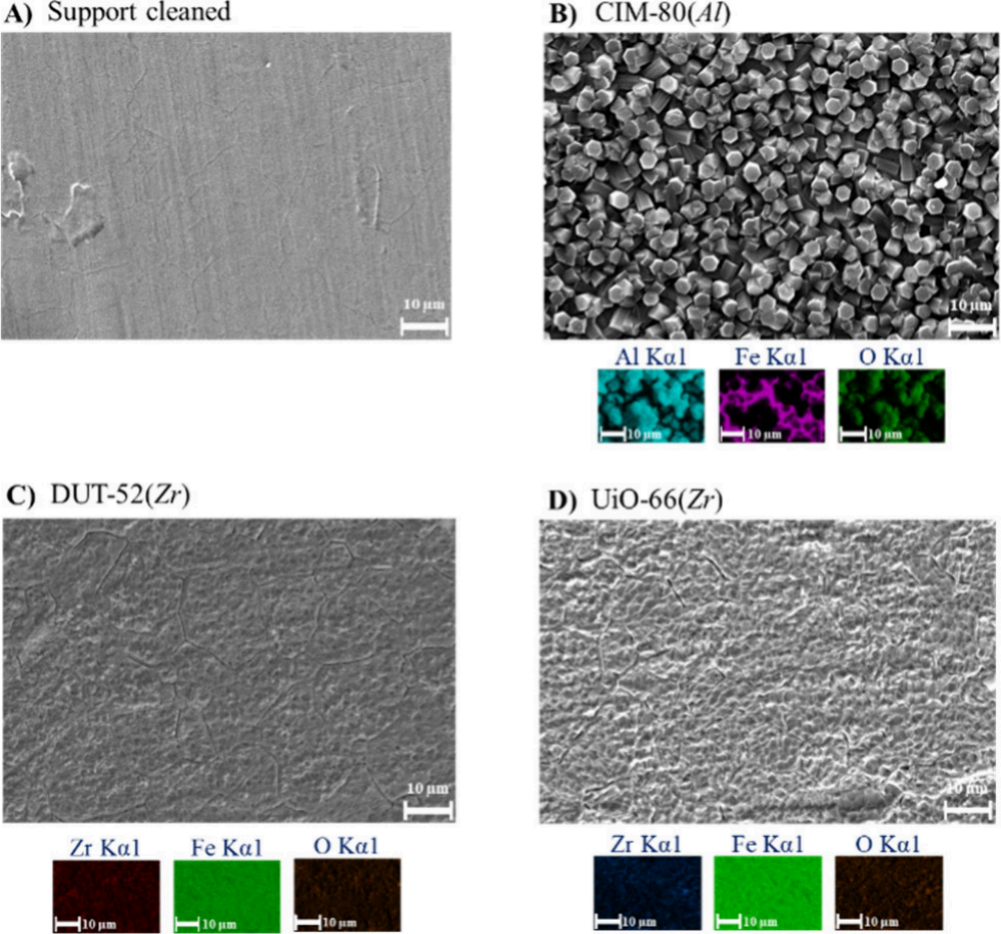
Characterization of the developed MOF-coated blades (SEM and EDX studies). (A) Support cleaned, (B) CIM-80 (*Al*), (C) DUT-52­(*Zr*), and (D) UiO-66 (*Zr*) MOFs.

SEM characterization of the further MOF-coated blades indicates two different results. Thus, a homogeneous layer of crystallites 2–10 μm in size was observed for CIM-80­(*Al*) (Figure 3B and Figure S3). However, individual crystallites were not observed in the coatings of DUT-52­(*Zr*) and UiO-66­(*Zr*), indicating a nanometer size or epitaxial growth (Figure 3C,D and Figure S3). This could not be confirmed by PXRD due to the extremely thin layer of MOF, leading to a poor intensity–background ratio for the X-ray reflections. The formation of a homogeneous layer of DUT-52­(*Zr*) and UiO-66­(*Zr*) was in any case also supported by the EDX measurements, indicating the presence of Zr in the layer (Figure 3C,D and Figure S4). Further details on the coatings can be observed in Figure S5 as well as in the characterization data summarized in Table S2. Although thickness was higher with the CIM-80­(*Al*)-coated blade, this is not the main aspect that will be further affecting the microextraction efficiency, as other aspects related to the MOF’s nature are also involved. These three MOFs are stable in water at different pH values and when exposed to complex biofluids for 24 h (urine, but also saliva and plasma). DUT-52­(*Zr*) is stable in urine for the time-lapse of the experiments proposed here, but it loses structural correlation in basic media at longer times (Figure S6).

### Influence of Setup Variables

The influence of the relevant instrumental setup variables in CBS-HRMS experiments was evaluated with the incorporation of the MOF-coated blades. All variables related to the desorption/ionization step were optimized using CIM-80­(*Al*)-coated blades, and the optimized conditions were then used with the remaining MOF-based coatings. Besides, four challenging polar compounds were selected as target analytes for this optimization. This way, 5 μL of a 1 mg·L^–1^ MeOH/H_2_O (1:1, *v*/*v*) solution containing the four targets was directly added on the outermost tip of the coated blade. Readily after this, 50 μL of the elution solvent was added on the same spot, and the CBS experiment was conducted immediately. The solvents evaluated in this procedure were water, ACN, MeOH, and IPA, as well as several mixtures, all of them with 0.1% (*v*/*v*) formic acid as an additive to enhance the ionization efficiency.

The high surface tension of water (72.8 mN·m^–1^) and ACN (30.2 mN·m^–1^) created a deformed droplet instead of a proper jet spray, leading to potential arching effects. IPA (21.7 mN·m^–1^) and MeOH (23.8 mN·m^–1^) have lower surface tensions, leading to a correct jet formation and allowing a reduction of the distance to the MS inlet, thus increasing the overall sensitivity of the method. Figure S7 shows that MeOH provided the best desorption/ionization of the analytes, which might be due to its intermediate surface tension ensuring proper jet formation. Then, different solvent mixtures based on MeOH were prepared to test if mixed solvents present any additional interesting properties. However, both ACN:MeOH (1:1, *v*/*v*) and IPA:MeOH (1:1, *v*/*v*) presented the same drawbacks as neat ACN and IPA, generating droplet irregularities and poor desorption performance, respectively (Figure S8). For that reason, MeOH with 0.1% formic acid was selected as the optimum solvent for further experiments.

In all these studies leading to the selection of MeOH, the voltage was evaluated in the range of 2.5–5.0 kV in 0.5 kV steps, fixing the distance on each variation to ensure a correct measurement, while minimizing arching from the blade to the MS inlet (Figure S7). Voltages below 3 kV were not enough to obtain a stable measurement, which might be related to the inability to overcome the surface tension of the solvent and generate a proper spray jet. On the other hand, 4.0 kV resulted in the best voltage for all analytes selected, except for COT, where 5.0 kV performed the best. Therefore, MeOH (with 0.1% (*v*/*v*) formic acid) as solvent and a 4.0 kV voltage were selected as optimal conditions for the desorption/ionization step.

### MOF-Coated Blade Selection

Once the optimum desorption solvent was selected, the extraction time was fixed to 3 min to ensure a fast method. Thus, the performance of the three MOF-coated blades, CIM-80­(*Al*), UiO-66­(*Zr*), and DUT-52­(*Zr*), was evaluated by immersion in a 1.5 mL aqueous standard of 2 mg L^–1^ for 3 min, followed by drying under a gentle N_2_ stream and further desorption/ionization using 50 μL of MeOH (with 0.1% (*v*/*v*) formic acid) for the CIM-80­(*Al*)-coated blade and 40 μL for the UiO-66­(*Zr*)- and DUT-52­(*Zr*)-coated blades. The decrease of the desorption volume for the Zr-MOF coatings is due to the droplet–MOF interaction, as it highly depends on the hydrophobicity. Thus, the Zr-MOF coatings favored the formation of arching effects (Figure S9).

The enrichment factors (E_F_) and extraction recoveries (E_R_) were calculated as detailed in the Supporting Information (Calculations S1) and are shown in Table 1 (attained under the optimum conditions), by direct comparison of the peak areas obtained with direct blade spiking and extraction of an aqueous standard with a MOF-coated blade, with contents prepared to ensure the same injected concentration in the MS (if 100% efficiency in extraction and/or desorption).

**Table 1:** Performance of the Method as E_F_ and E_R_ (%) Values Obtained for the Different MOF-Coated Blades

	CIM-80(*Al*)		UiO-66(*Zr*)		DUT-52(*Zr*)	
Analyte	E_F_	E_R_ (%)	E_F_	E_R_ (%)	E_F_	E_R_ (%)
CAF	3.0	9.9	1.4	3.7	3.4	9.2
NAP	18	60	11	29	7.9	21
COT	0.84	2.8	1.7	4.6	2.0	5.4
ACE	0.40	1.3	0.3	0.83	2.0	5.2

E_F_ can be calculated as the quotient between the peak area obtained by the extraction experiment and the peak area obtained by direct blade spiking. E_R_ is calculated by the quotient between E_F_ and E_Fmax_, this being the maximum preconcentration attainable. This value estimation has been carried out considering that all of the desorption solvent enters the MS inlet. However, real experimental conditions and factors (solvent surface tension, surface polarity, and sorbent) might lead to a lower E_Fmax_. All in all, E_Fmax_ is estimated to be 30 for CIM-80­(*Al*)-coated blade and 38 for both Zr-MOF-coated blades (considering 1.5 mL is the sample volume and 40–50 μL are the respective desorption solvents).

The CIM-80­(*Al*)-coated blade outperforms the remaining blades, in terms of E_F_ and E_R_, for the determination of NAP, achieving impressive results of 18% and 60%, respectively. This is highly satisfactory considering that most microextraction approaches are nonexhaustive and the main purpose is to attain a high preconcentration (E_F_) in short timing. Besides, the UiO-66­(*Zr*)-coated blade shows satisfactory results for NAP and COT, obtaining E_F_ values of 11 and 1.7, respectively, and E_R_ values of 29% and 4.6%, respectively. On the other hand, the DUT-52­(*Zr*)-coated blade was the only one able to preconcentrate ACE while also showing the best results for COT. Besides, it was adequate for NAP and successful for CAF, with the latter being quite similar in performance to the CIM-80­(*Al*) device. Based on the results obtained, the DUT-52­(*Zr*)-coated blade was the one selected for further studies, intending to have a broad ability for extraction of analytes of a different nature.

Calibration curves were accomplished with aqueous standards of target analytes, subjected to the entire method. *d*_8_-CAR, *d*_6_-DIU, and *d*_3_-KET were incorporated as deuterated surrogates to minimize the signal fluctuation related to ambient measurements. Table 2 lists the main analytical figures of merit of the calibrations obtained, including linearity, the method limits of detection (LODs) and quantification (LOQs), estimated as the lowest concentration that provides a detectable signal and the lowest concentration that ensures a relative standard deviation (RSD) and relative error (RE) lower than 30% and 40%, respectively. Besides, each calibration was carried out with two different blades, and therefore, the reproducibility achieved with the calibration also accounted for interdevice precision, fulfilling the criteria of common European method development regulation.^33^ Table S3 includes a specific study carried out to evaluate the device-to-device reproducibility involving the target analytes. Moreover, RSD values for intraday precision in both LC-MS aqueous standards and with standards in synthetic urine, at low (40 ng·mL^–1^) and midlevel concentrations (500 ng·mL^–1^), were determined. The calibration range was between 10 and 2000 ng·mL^–1^, considering different linear sections due to the wide concentration range defined. For all of the calibrations, the determination coefficients ranged between 0.992 and 0.998. With regard to the sample volume required (300 μL), it is comparable and even lower than others described in CBS-HRMS studies from the literature (Table S4). Adequate RSD values were achieved, ranging from 8.8% for CAF to 26% for COT at low concentration and 1.0% for NAP to 30% for COT and CAF at medium concentration. LOQs ranged between 0.1 ng·mL^–1^ for CAF and 40 ng·mL^–1^ for NAP, thus supporting the applicability of the devices for ultratrace screening analysis. These LOQs are similar to those obtained with other devices developed for CBS applications with biofluids and food samples (Table S4), such as HLB,^34,35^ HLB derivatives,^14,19,36^ and other composites,^25,37^ but in the current study, these LOQs are achieved within a very short time. In fact, all these literature studies involve extraction times from 10 min to 20 min (Table S4), but the MOF-coated blades developed in this study require only 3 min while ensuring acceptable accuracy levels in the range of 73% to 133%. Additionally, sustainability and practical metrics were calculated using the SPMS^38^ and BAGI systems,^39^ achieving score values of 7.89 out of 10 and 62.5 out of 100, respectively, showing the greenness and high-throughput of the CBS­(MOF)-HRMS screening methodology (Figure S10).

**Table 2:** Main Figures of Merit Obtained by the Developed Methodology with MOF-CBS Devices

							Repeatability Intraday (RSD, %)		
							Aqueous Standards		Synthetic Urine Standards
Analyte	Calibration Range (ng·mL^–1^)	(Slope ± Uncertainty) × 10^–3^	R^2^	Standard Deviation of Residuals	LOD (ng·mL^–1^)	LOQ (ng·mL^–1^)	Low Level^a^	Mid Level^b^	Mid Level^b^
CAF	0.100–100	5.5 ± 0.5	0.996	1.5 × 10^–2^	0.03	0.1	8.8	9.4	30
	100–1000	3.2 ± 0.6	0.996	9.8 × 10^–2^					
NAP	40–1000	0.20 ± 0.03	0.996	6.8 × 10^–3^	12	40	25	4.1	1.0
COT	10–100	11 ± 2	0.998	2.4 × 10^–2^	3.3	10	26	30	22
	100–1000	3.4 ± 0.9	0.992	1.5 × 10^–1^					
ACE	20–1000	0.40 ± 0.04	0.996	1.1 × 10^–2^	6.0	20	12	11	17

a Low level of concentration: 40 ng·mL^–1^.

b Mid level of concentration: 500 ng·mL^–1^.

### Analysis of Urine Samples

#### Target Analysis

Ten urine samples (coded from U01 to U10) were analyzed under optimal conditions with the DUT-52­(*Zr*)-coated blades. As shown in Figure 4, CAF was the native target analyte most frequently detected, being found in 8 out of the 10 urine samples, with concentrations ranging from low ng·mL^–1^ values (13 ng·mL^–1^) in the case of sample U04 up to 3670 ng·mL^–1^ for sample U02.

**Figure 4:**
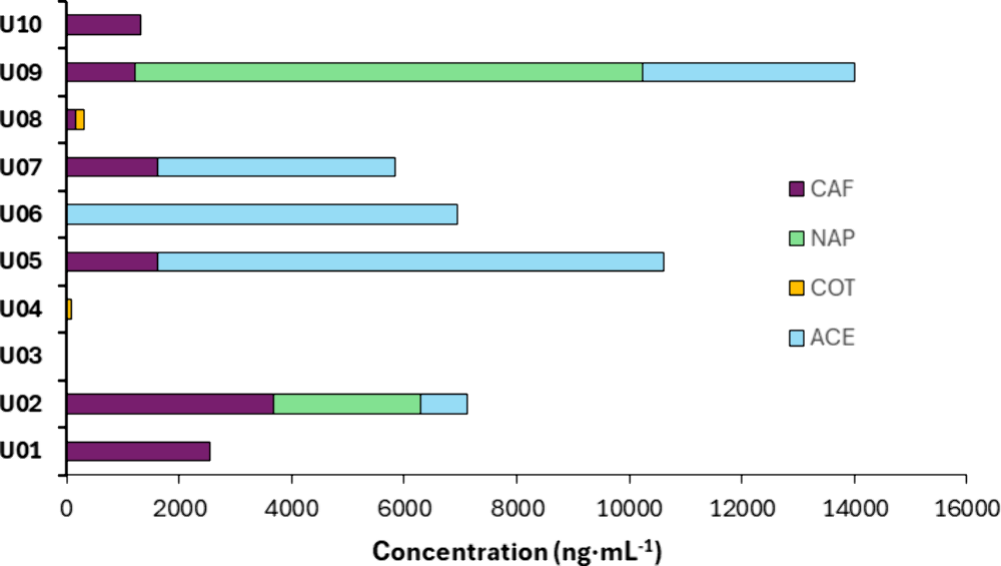
Concentration of analytes (in ng·mL^–1^) found in real urine samples of healthy volunteers using the DUT-52­(*Zr*)-coated blades in CBS-HRMS.

Among all samples, 87.5% of patients mentioned a weekly or daily basis consumption of caffeine-based drinks (2–3 cups per day), whereas 12.5% commented on a consumption of 1–2 times per week. COT was also found at low concentrations in 2 samples. These concentration levels could be directly correlated to smoker patients due to the metabolism of nicotine to cotinine. Thus, surveyed daily smokers showed concentrations of cotinine of 81 and 145 ng·mL^–1^ for U04 and U08, whereas nonsmokers did not show detectable concentrations of COT. On the other hand, NAP, an anti-inflammatory drug, was quantified in sample U09 at a relatively high concentration (9009 ng·mL^–1^) requiring further dilution for proper quantification. This level could be associated with the profile of a young woman with a frequent use of pain killers, as NAP is also used to treat menstrual pain.

Finally, ACE, a well-known analgesic and antipyretic, was identified in 50% of the samples (Figure 4), and indeed, the consumption of anti-inflammatory drugs was mentioned by 50% of the volunteers in the survey. In any case, endogenous compounds with the same exact mass as the exogenous substance could lead to false positives when using AIMS for the analysis of biofluids. This is the case for ACE, which presents a mass interference with the endogenous compound dopamine quinone. Because of that, it could only be semiquantified using the CBS-HRMS approach. In particular, it was semiquantified only in the samples where the specific [C_6_H_8_NO]^+^ product ion (110.0600 *m*/*z*) was observed in the MS/HRMS spectrum, this way avoiding false positives. Thus, the concentration of ACE in the samples ranged from 3769 to 9005 ng·mL^–1^. Moreover, glucuronide metabolites of NAP, COT, NAP, and ACE were also monitored considering the biological nature of the samples. Thus, it was possible to detect NAP-Glu in U02, exhibiting the applicability of the designed devices for a wider spectrum of analytes that could help to ensure proper identification.

#### Suspect Screening Analysis

As mentioned before, suspect and nontarget screening are challenging strategies to accomplish simultaneously, given the necessity of a fast analysis and the lack of chromatographic separation, which certainly is difficult for data acquisition and data analysis, especially with biofluids. For these reasons, this simultaneous aim has been scarcely explored.^40–43^ Thus, this work carefully optimizes the acquisition and data analysis workflow to develop a reliable suspect screening approach using CBS (with MOF-coated blades)-HRMS (described in detail in Procedure S1). To do so, the data acquisition was automated to improve the throughput and performance of the analyses. During each analysis, the sample was measured in triplicate, modulating the spray voltage from 0 to 4.0 kV in time events of 1 min (as shown in Figure S11). As the ionization/desorption step is not exhaustive, it is possible to carry out consecutive desorption steps (replicates) in the same sample, although a cleanup is required after analysis to avoid carryover if the MOF-coated blades want to be reused. Briefly, 40/50 μL of MeOH (0.1% HCOOH) was deposited onto the MOF-coated blade every time the spray voltage was set at 0 kV, and then the spray voltage was increased up to 4.0 kV to allow the desorption/ionization of the analytes from the MOF. The 1 min window events were set after careful optimization to ensure both the complete spraying of the elution solvent and the safety operation for the loading step. This automated approach permitted us to increase the reliability of the suspect screening analysis, as more MS/HRMS spectra could be acquired for a sample in a single run.

Moreover, data analysis can also be a bottleneck, as the generated data do not follow a Gaussian chromatographic peak distribution, thus hiding the peak detection by the Compound Discoverer software (Thermo Scientific), complicating the use of suspect screening workflows already developed for LC-HRMS analysis. To sort out this issue, the peak rating, which is a parameter involving different chromatographic quality shape criteria to filter out low quality peaks, was set to 0. Additionally, all features within a time frame of 1 min presenting the same *m*/*z* value were merged to simplify data treatment, as this is the time set for the voltage pulses. Using this approach, each feature could be detected up to 3 times per run, increasing the confidence in the identification. Clearly, the absence of chromatography when using AIMS may complicate the tentative identification of compounds since retention prediction models cannot be applied to increase the confidence in the annotations. Thus, an identification workflow was also proposed (Procedure S3) based on that of Musatadi et al.^44^ According to these authors, a mass list containing common endogenous compounds in urine was used as an inclusion list. This ensures a double check of those features where both exogenous and endogenous compound identifications could be plausible, therefore minimizing false positive or negative identifications. As an example, Figure S12 shows the MS/HRMS spectra of exogenous/endogenous pair cotinine/serotonine (177.1021 *m*/*z*), where the tandem mass spectra confirmed the annotation of this compound as the exogenous cotinine.

In total, 132 compounds were identified (Table S5) when the developed workflows were applied for data acquisition and analysis. Among them, 43 compounds were tentatively annotated with a high confidence level (1–3) in the Schymanski scale^45^ (Figure 5). 44.2% of the annotations correspond to endogenous substances that are easily identified due to their higher concentration in biofluids. On the other hand, 41.9% of the compounds were tentatively identified as exogenous substances (15 out of 18 with a confidence level higher than 3), while 14% of the annotations belong to endogenous/exogenous chemical pairs, which could not be annotated with a confidence level higher than 3. Among the exogenous compounds, personal care products (28%), medical drugs (24%), and industrial chemicals (20%) were the compounds most frequently found. This can be correlated with the daily use of cosmetics, skin creams, hair and body wash, as well as anti-inflammatory drugs, pain killers, and even contact with plasticizers. Additionally, to a lesser extent, it was also possible to tentatively identify drugs of abuse (8%) such as anthranilic acid, pesticides (4%) like pyroquilon, food biomarkers (8%) associated with wheat and grape consumption, and even fragrances (4%) and UV filters (4%) such as methyl ricinoleate and bemotrizinol, respectively.

**Figure 5:**
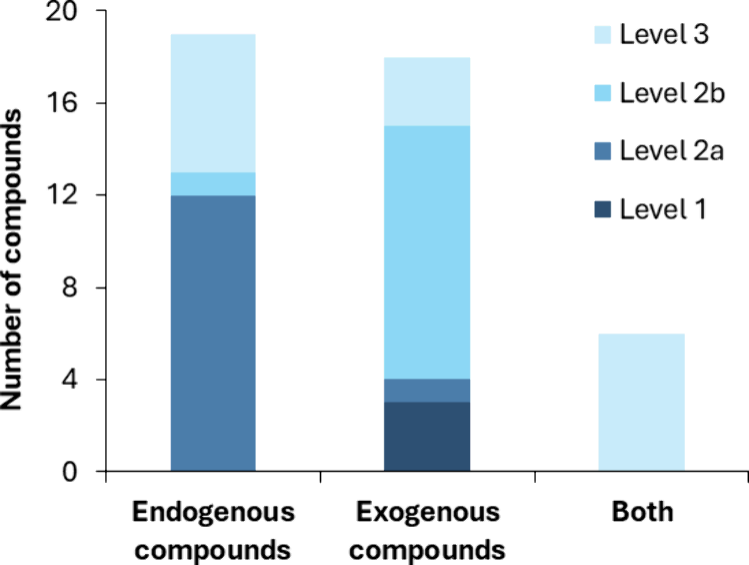
Compounds tentatively identified (confidence level of 1–3) with a CBS­(MOF)-HRMS suspect screening approach.

## Conclusions

This study has shown a novel approach for CBS bioanalysis based on the incorporation of MOFs. This article has presented a simple protocol for the preparation of MOF-coated blades by a mild thiol-based functionalization strategy, with proven stability of the resulting devices, successful incorporation of the crystalline material onto the blades, and geometry and dimensions that are fully compatible with robotic autosamplers for parallel sample preparation in the 96 well format. Besides, not only has the resulting MOF-based CBS been demonstrated to be a suitable alternative for targeted analysis, characterized for short timing and high efficiency, but also its applicability to suspect screening analysis has been proven. The CBS with MOF-coated blades and HRMS succeeded in the simultaneous identification of a wide variety of both endogenous and exogenous species in complex biological samples with contrasting results. In addition to this, an entire workflow was successfully developed to permit the simple incorporation of suspect screening in CBS procedures parallel to target analysis, drastically increasing the value of the studies not only for exposomic but also for metabolomic applications. This proof of concept will serve as a first step in the field of high-throughput biomonitoring in human biofluids.

## Supplementary Material



## Notes

Chemical and reagents; suspect screening workflow for MOF-based coatings in CBS-HRMS; procedure for PXRD and FTIR analyses; assignment of confidence levels using CBS-HRMS; estimation of enrichment factors; additional characterization data including FTIR spectra, SEM images, PXRD patterns, desorption solvent data, sample photographs and contact angles, and MS/HRMS spectra; score metrics of CBS­(MOF)-HRMS for sustainability and practicality; voltage-pulsed acquisition to increase both repeatability and automatization; synthetic conditions to prepare MOF coatings; elemental composition of MOF-coated blades; interdevice reproducibility; main features of methods involving CBS-HRMS; and compounds tentatively identified in urine samples by CBS­(MOF-coated blades)-HRMS (PDF)
