# Supplementary material for: Exploring Metal–Organic Framework-Coated Blades for Direct and High-Throughput Screening Analysis of Complex Biological Matrices
Source: Anal Chem. 2025 Aug 20;97(34):18621–9. doi: 10.1021/acs.analchem.5c02782 (PMC12409693; doi:10.1021/acs.analchem.5c02782)
Supplement: Supplementary file 1 [file ac5c02782_si_001.pdf]

Supporting information

Exploring metal-organic framework-coated blades for direct and high-throughput screening analysis of complex biological matrices

Isaac Negrín-Santamaría[1,2], María J. Trujillo-Rodríguez[1,2], Encarnación Moyano[3], Juan H. Ayala[1], Olatz Zuloaga[4,5], Jorge Pasán[6], Verónica Pino[1,2,7]*, Juan F. Ayala-Cabrera[4,5]*

[1]Laboratorio de Materiales para Análisis Químico (MAT4LL), Departamento de Química, Unidad Departamental de Química Analítica, Universidad de La Laguna (ULL), Spain.

[2]Unidad de Investigación de Bioanalítica y Medioambiente, Instituto Universitario de Enfermedades Tropicales y Salud Pública de Canarias (IUETSPC), ULL, Spain.

[3]Department of Chemical Engineering and Analytical Chemistry, University of Barcelona (UB), Barcelona, Spain.

[4]Department of Analytical Chemistry, University of the Basque Country (UPV/EHU), Leioa, Spain.

[5]Research Centre for Experimental Marine Biology and Biotechnology (PiE-UPV/EHU), University of the Basque Country, Plentzia, Spain

[6]Laboratorio de Materiales para Análisis Químico (MAT4LL), Departamento de Química, Unidad Departamental de Química Inorgánica, Universidad de La Laguna (ULL), Spain.

[7]Centro de Investigación en Red de Enfermedades Infecciosas (Ciberinfec), Instituto de Salud Carlos III, Spain

INDEX

Experimental

Experimental S1 .... Page S3
Procedure S1 (including Figure ProcedS1 and Table ProcedS1) .... Page S4
Procedure S2 .... Page S7
Procedure S3 (including Figure ProcedS3) .... Page S8

Calculations

Calculations S1 .... Page S10

Figures

Figure S1 .... Page S11
Figure S2 .... Page S13
Figure S3 .... Page S14
Figure S4 .... Page S15
Figure S5 .... Page S16
Figure S6 .... Page S17
Figure S7 .... Page S20
Figure S8 .... Page S21
Figure S9 .... Page S22
Figure S10 .... Page S23
Figure S11 .... Page S24
Figure S12 .... Page S25

Tables

Table S1 .... Page S26
Table S2 .... Page S27
Table S3 .... Page S28
Table S4 .... Page S29
Table S5 .... Page S32

Experimental S1. Chemicals and Reagents.

Stainless steel sheets were purchased from Bonnet (Tenerife, Spain) for the preparation of MOF-coated blades. Nitric acid (96%), sulfuric acid (96%), and sodium hydroxide (99%), used for the surface treatment, were supplied by Honeywell (Indiana, USA), Scharlab (Barcelona, Spain) and Sigma-Aldrich (Steinheim, Germany), respectively. Surface functionalization was performed with mercaptoacetic acid (98%), supplied by Sigma-Aldrich. MOF precursors and reagents, mesaconic (99%), 2,6-naphtalenedicarboxilic (95%) and terephthalic (98%) acids, aluminum (III) nitrate nonahydrate (98.5%), zirconium (IV) chloride anhydrous (98%), urea (99%), *N,N*-dimethylformamide (DMF), hydrochloric and acetic acid were bought from Sigma-Aldrich. MOF-based devices were prepared by solvothermal synthesis in 23 mL Teflon-lined reactors and stainless-steel autoclaves provided by Anton Parr (Ashland, VI, USA). Methanol (MeOH) and ultrapure water, both with LC-MS grade, were purchased from Panreac AppliChem (Barcelona, Spain), while acetonitrile (ACN) and LC-MS grade 2-propanol (IPA) were provided by Scharlab (Barcelona, Spain) and Avantor (PE, USA), respectively. Formic acid (99.9%), supplied by Sigma-Aldrich, was added to all elution solvents to enhance ionization efficiency. For the attachment of blades to the system, copper alligator tweezers, obtained from Muller Electric (Akron, OH, USA), were used.

Procedure S1. Suspect screening workflow for MOF-based coatings in CBS-HRMS.

The following workflow was developed for the suspect screening of xenobiotics in human urine using a CBS(MOF)-HRMS approach. The acquisition consisted of a full scan combined with a data dependent acquisition, as explained in section 2.4 of the main manuscript. The workflow is summarized in the following **Figure ProcedS1**.

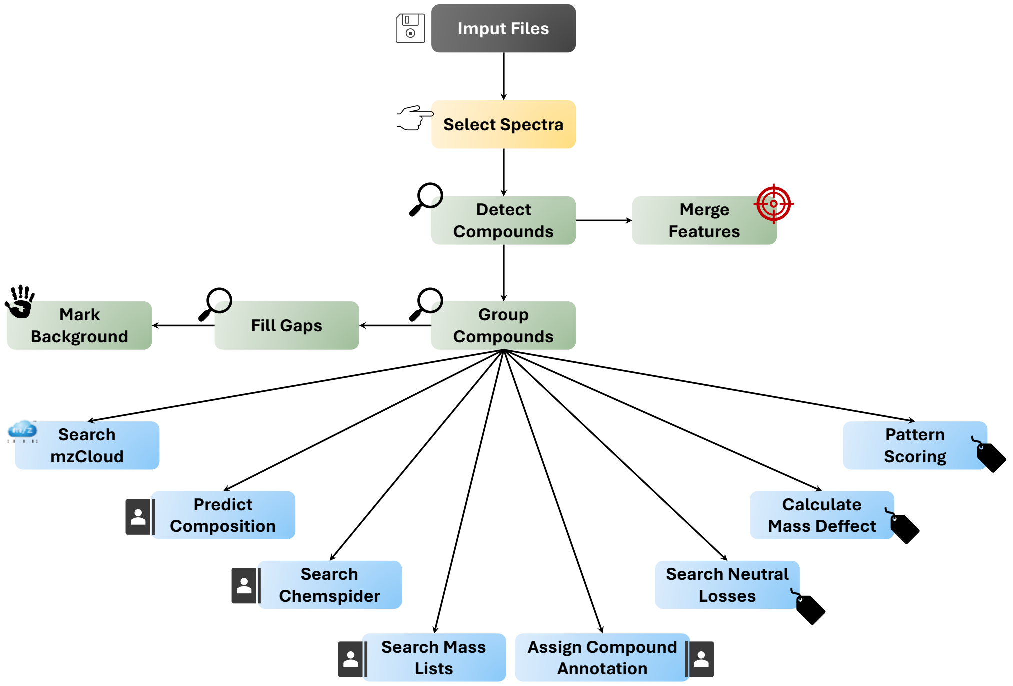

Figure ProcedS1. Suspect screening workflow using the Compound Discoverer 3.3 Software (Thermo Scientific).

The first nodes are related to the loading of the files and the selection of the spectra along the whole chronogram (0-5.5 min). After that, the ***Detect Compounds node*** aims to look for unique features in the sample. The following restrictions were applied: (i) only the most intense isotope was chosen except for specific element isotope patterns (i.e., Br and Cl), (ii) the peak detection signal-to-noise (S/N) ratio was set to 0 as there is no chromatographic peak, and (iii) $[M+H]^+$, $[M+NH_4]^+$ and $[M+Na]^+$ formulas were selected as potential ion definitions. As there is no chromatographic separation and a pulsed acquisition was optimized (**Figure S11**), a mass tolerance of 5 ppm and a retention time tolerance of 0.95 min were set in the ***Merge Features node***. The ***Group Compounds node*** aligns features between different samples which show the same retention time (± 0.5 min), the same *m/z* value (± 5 ppm), and the same ion formula. Additionally, as explained in section 3.4 (subsection: *Suspect Screening Analysis*), all parameters related to the peak rating were set to 0, to avoid false negatives due to a bad peak shape consideration. The Fill Gaps node provides a value for those features with a S/N lower than 1.5 whereas the Mark Background node highlights those features with a sample/blank ratio lower than 5. All the remaining nodes are related to the feature annotation steps. Thus, they are integrated in the ***Assign Compound Annotations node*** where the different data sources are prioritized in the following order: (i) predicted composition, (ii) mzCloud search, (iii) mass list search, and (iv) ChemSpider search. The specific details for each of these nodes are summarized in **Table ProcedS1**.

Table ProcedS1. Specific parameters of nodes, related to the assignment of the compound annotation.

Predict Compositions Node	
**Parameter**	**Value**
1. Prediction settings	
Mass tolerance	5 ppm
Min. Element	C H
Max. Element	C90 H190 Br3 Cl8 F60 N10 O18 P3 S5
Min. RDBE	0
Max. RDBE	4
Min. H/C	0.1
Max. H/C	3.5
Max. Candidates	10
2. Prediction settings	
Intensity tolerance	30%
Intensity threshold	0.1%
S/N threshold	3
3. Fragments Matching	
Use it	True
Mass tolerance	5 ppm
S/N threshold	3

Search mzCloud Node	
**Parameter**	**Value**
1. General settings	
Compound classes	All
Library	Autoprocessed; Reference
Search MSn Tree	False
2. DDA Search	
Identity Search	Cosine
Match activation type	True
Match activation energy	Match with tolerance
Activation energy tolerance	20
Apply intensity threshold	True
Match factor threshold	30

Search Mass List Node	
**Parameter**	**Value**
1. Search settings	
Mass lists	Metabolites, Parents and Endogenous mass lists
Use Retention time	False
Mass tolerance	5 ppm

Search ChemSpider Node	
**Parameter**	**Value**
1. Search settings	
Databases	ChEBI, EPA DSSTox; EPA Toxcast; KEGG; NIAID
Search mode	By formula or mass
Mass tolerance	5 ppm
Max. results per compound	100

Additionally, other nodes were also included to enhance the confidence on the annotations. The ***Search Neutral Losses node*** looks for specific neutral losses of certain families. In this work, it was set to identify potential phase II metabolites by monitoring glucuronide neutral losses ($C_6H_8O_6$, 176.03 Da). The ***Pattern Scoring node*** looks for specific patterns related to Cl- or Br-containing compounds. Finally, the ***Calculate Mass Defect node*** looks for features with a repeating unit such as $-CF_2-$ or $-C_2F_3O-$ for PFAS or $-CH_2-$ for compounds with the same functional group and different aliphatic chains.

Procedure S2. PXRD and FTIR analyses.

The PXRD analysis of these coated blades is complex, beginning with the proper X-ray diffraction experiment itself. A special holder case was 3D printed for the blades, in such a way that the coating will be at the exact height to maximize diffraction (i.e. the thickness of the stainless-steel piece has to be subtracted to align the system).

In general, the more MOF material on the blade surface, the better will be the PXRD data, as it occurs with the CIM-80(*Al*) MOF. The coatings of DUT-52(*Zr*) and UiO-66(*Zr*) are much thinner, and the characteristic peaks of the MOF hardly overcome the X-ray dispersion by the blade (be aware that the stainless-steel diffraction peaks appear at higher 2-theta values than registered in this analysis). Being so thin, and without individual crystals on the coating, it is possible that the growth of the MOF in these two cases is epitaxial, that implies that not all the characteristic peaks of the MOF will be present in the PXRD, only those corresponding to the planes grown.

Considering all this, it is accepted as a good indication for the formation of the MOF the presence of the first characteristic peak (which is usually the most intense). In DUT-52(*Zr*) and UiO-66(*Zr*) this first peak is much more intense than the others, even in the bulk material, the presence of this peak in the PXRD of the blade, together with the presence of Zr in the EDX, is considered support enough for the formation of the MOFs.

With respect to FTIR characterization, it was conducted with two different approaches. First, MOF powder characterization was conducted placing few milligrams of the freshly synthetized and dry material in the FTIR instrument. Then, they were pressed down by the swivel press to compact the powder material to the glass, and then the IR spectra were collected. MOF-blades were characterized in a similar way, by pressing the device with the press to ensure the contact with the glass, allowing the proper measurement of the different crystalline materials grown onto the surface. The noise present in the spectra of the blades is coming from the thin air layer between the ATR crystal and the blade. This way, only the most relevant wavenumber range of the spectra is shown.

Procedure S3. Assignment of confidence levels using CBS(MOF-coated blades)-HRMS.

The following workflow was applied to assign the confidence on the tentative annotations proposed for the identification of endogenous and exogenous compounds in urine, using the CBS(MOF-coated blades)-HRMS approach (**Figure ProcedS3**). This workflow is based on that proposed by Musatadi *et al.* for the suspect screening of xenobiotics in biofluids by LC-HRMS[1]. First of all, the peak picking is carefully checked, and several filters are applied to sort out the list of features. These criteria include: (i) the presence of the feature in at least 2 consecutive desorption steps, (ii) a ratio sample/blank > 5, (iii) the acquisition of the MS/HRMS spectrum, and (iv) the ion formula is assigned with a mass error < 5 ppm and an isotopic pattern fit > 70%. After that, the workflow is applied, to classify the tentative annotations in different confidence levels, depending on: (i) the assignment of a chemical formula, (ii) the match with mzCloud spectral library and/or *in-silico* fragmentation spectra, and (iii) the match with a pure standard. As suggested by Musatadi *et al.*, a mass list containing endogenous compounds was included to avoid both type I and type II errors[1]. Since there is no retention time information when using CBS(MOF-coated blades)-HRMS, the differentiation of endogenous and exogenous isobaric compounds must be carried out by MS/HRMS spectra comparison.

(1) Musatadi, M.; Caballero, C.; Mijangos, L.; Prieto, A.; Olivares, M.; Zuloaga, O. et al. *Anal. Bioanal. Chem.* **2022**, *414*, 6855–6869. DOI:10.1007/s00216-022-04250-w

This is ref. 44 of the manuscript.

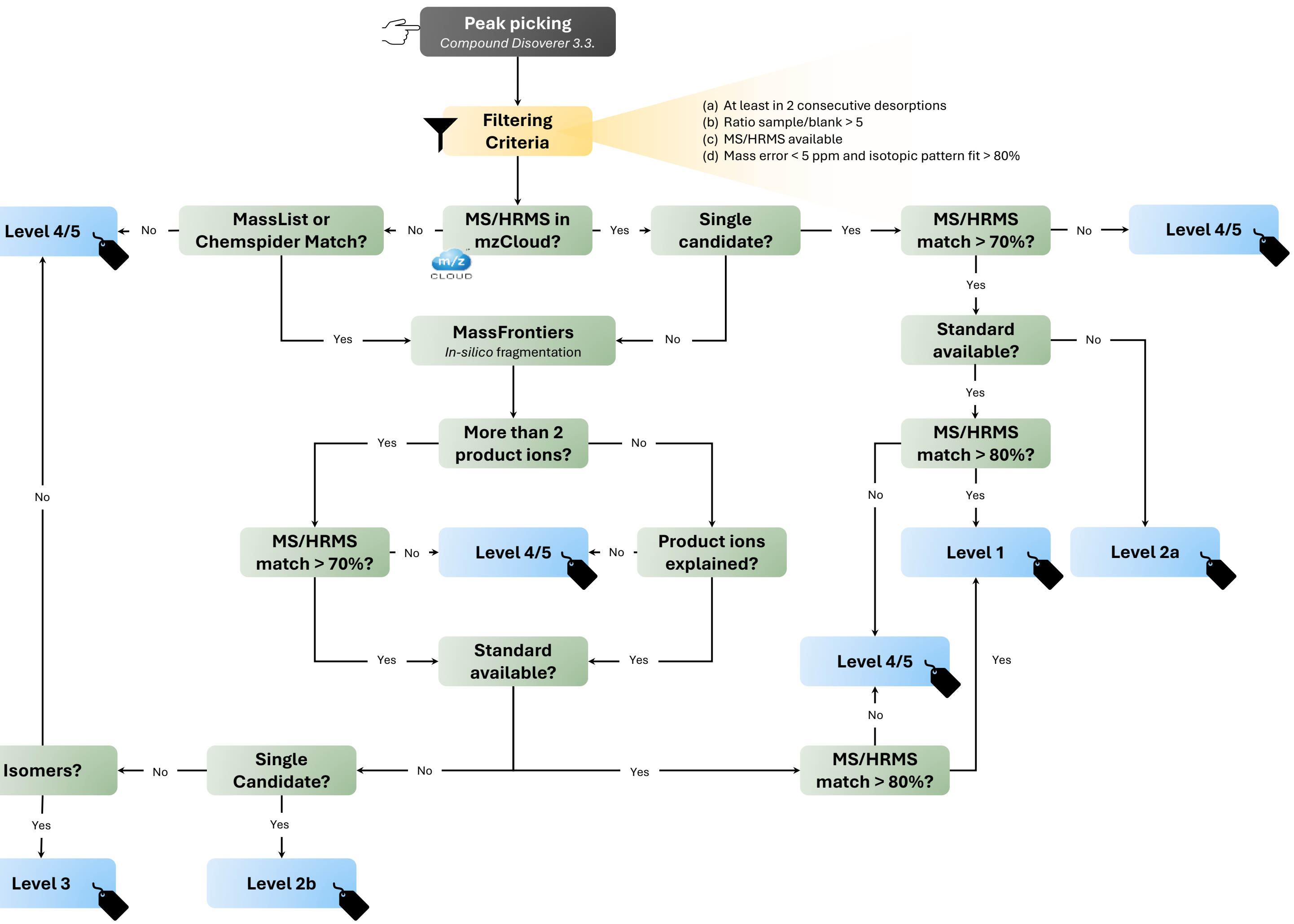

Figure ProcedS3. Proposed suspect screening workflow to assign confidence levels in ambient ionization mass spectrometry.

Calculations S1. Evaluation of enrichment factors, maximum enrichment factors, and extraction efficiencies.

*Estimation of the obtained enrichment factor (preconcentration factor).

Experiments are carried out immersing the MOF-based blades into an aqueous standard followed by desorption, together with experiments directly spiking the same amount of analytes (assuming 100% extraction efficiency) onto the MOF-based blades, also followed by desorption and MS analysis.

$$\mathbf{E_F} = \frac{Combined^a \ peak \ area \ of \ extractions \ with \ MOF - based \ blades}{Combined^a \ peak \ area \ of \ direct \ spike \ of \ MOF - based \ blades}$$

[a]Sum of peak areas obtained by three consecutive desorption.

*Estimation of the maximum enrichment factor.

This parameter assumes that all desorbed solvent-containing preconcentrated analytes totally reaches and enters the MS inlet:

$$\mathbf{E_{F_{MAX}}} = \frac{Volume \ of \ the \ sample/aqueous \ standard}{Volume \ of \ the \ desorption \ solvent}$$

where volume of the sample/aqueous standard for this study is fixed to 1.5 mL and, volume of desorption solvent are 0.05 mL for CIM-80(*Al*) and 0.04 mL for DUT-52(*Zr*) and UiO-66(*Zr*). Therefore, the E_{FMAX} values are 30.0 for CIM-80(*Al*) and 37.5 for DUT-52(*Zr*) and UiO-66(*Zr*).

*Estimation of the extraction efficiency.

It is calculated as in any microextraction strategy:

$$\mathbf{E_R}(\%) = \frac{E_F}{E_{F_{MAX}}} \times 100$$

Figure S1. FTIR spectra obtained with MOF powders and with the MOF-based blades. MOF powder characterization was conducted placing few milligrams of the freshly synthetized and dry material in the FTIR instrument. Then, they were pressed down by the swivel press to compact the powder material to the glass, and then the IR spectra were collected. MOF-blades were characterized in a similar way, by pressing the device with the press to ensure the contact with the glass, allowing the proper measurement of the different crystalline materials grown onto the surface. The noise present in the spectra of the blades is coming from the thin air layer between the ATR crystal and the blade. This way, only the most relevant wavenumber range of the spectra is shown. The Figures correspond to (**A**) CIM-80(*Al*), (**B**) UiO-66(*Zr*) and (**C**) DUT-52(*Zr*).

(**A**) CIM-80(*Al*)

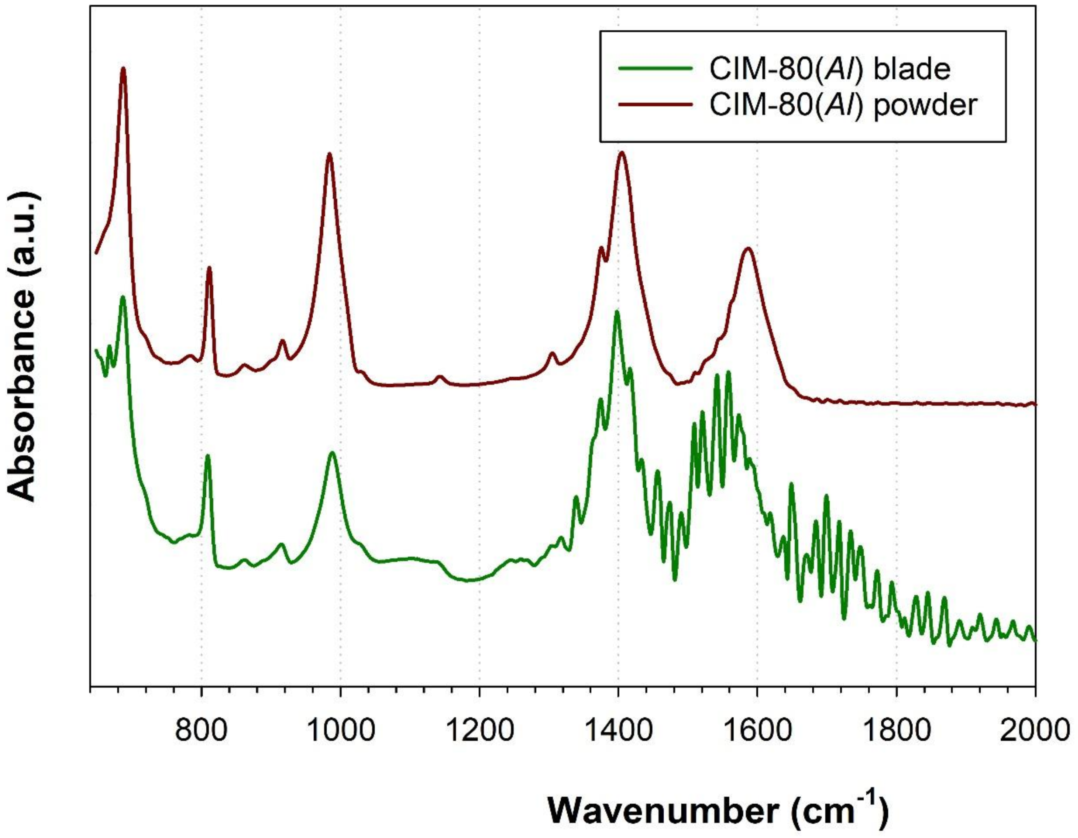

(**B**) UiO-66(*Zr*)

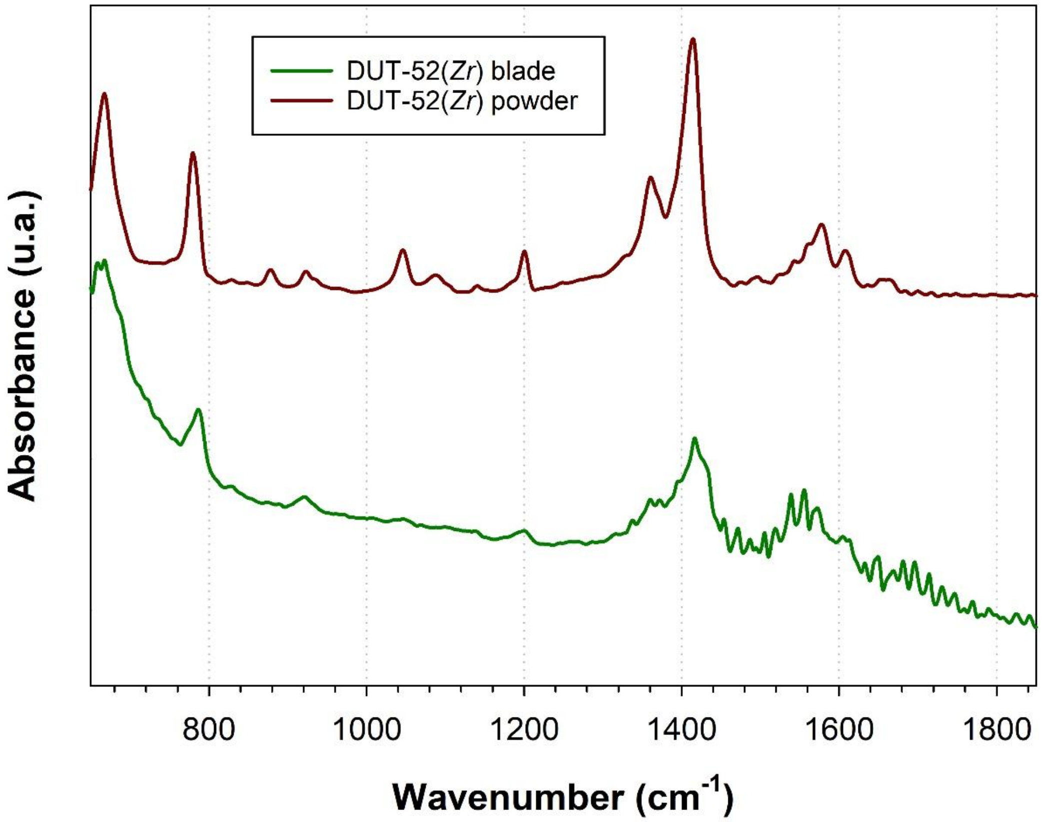

(**C**) DUT-52(*Zr*)

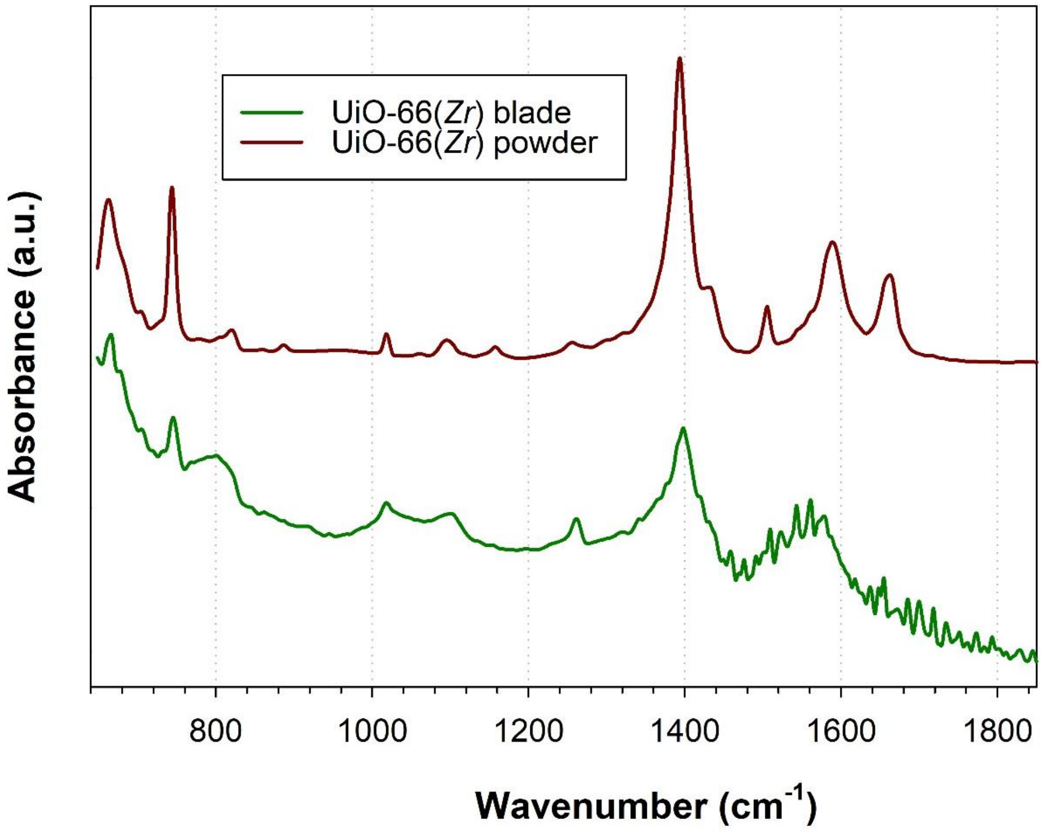

Figure S2. SEM characterization of a neat blade support treated with the optimum etching strategy.

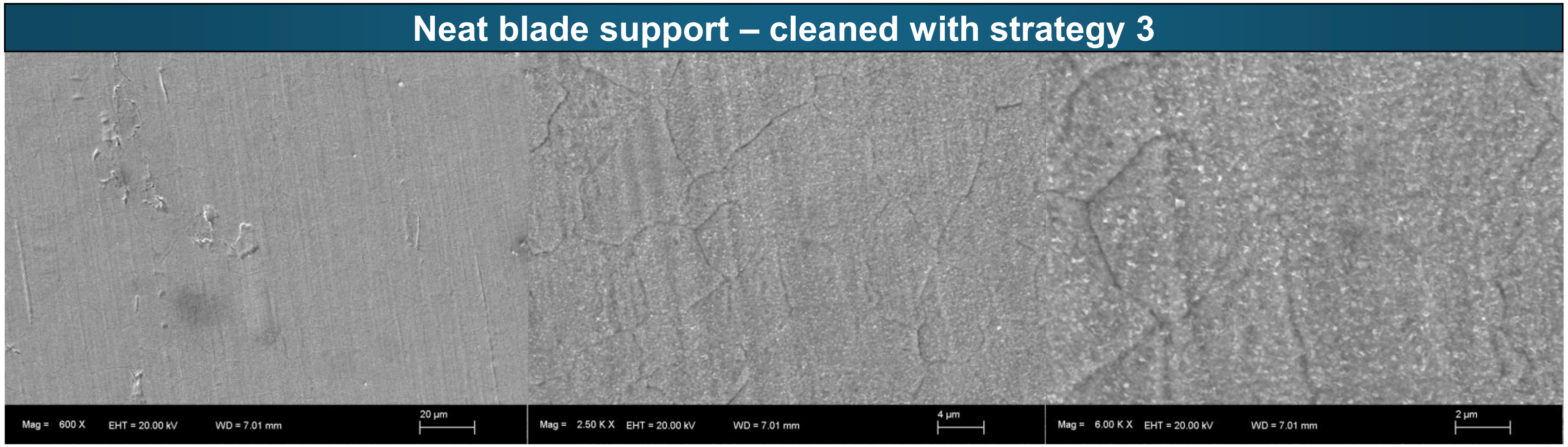

Figure S3. SEM characterization of the MOF-coated blades, being **A)** CIM-80(*Al*), **B)** DUT-52(*Zr*), and **C)** UiO-66(*Zr*).

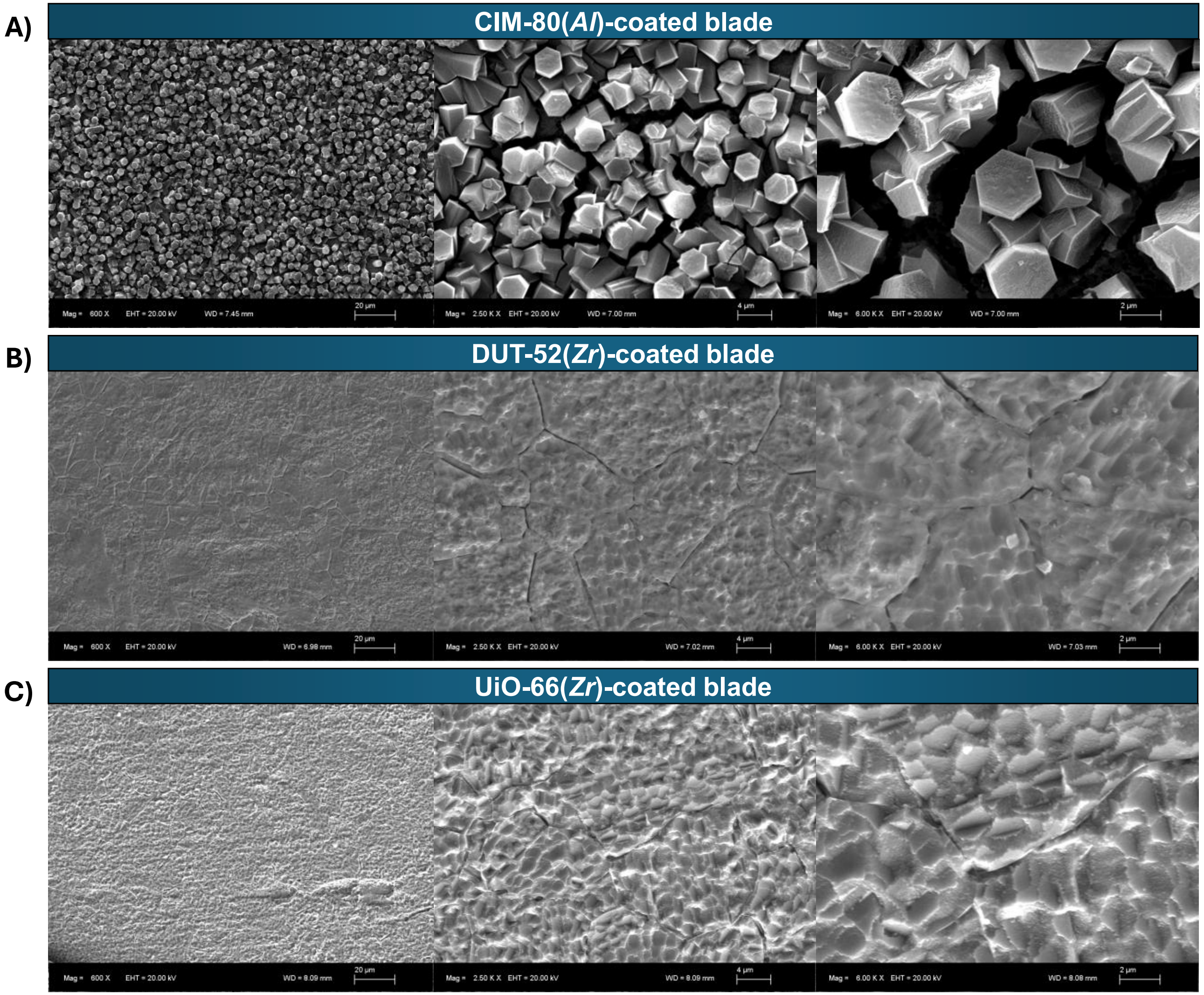

Figure S4. EDX mapping for **A)** CIM-80(*Al*)-coated blade, **B)** DUT-52(*Zr*)-coated blade, and **C)** UiO-66(*Zr*)-coated blade.

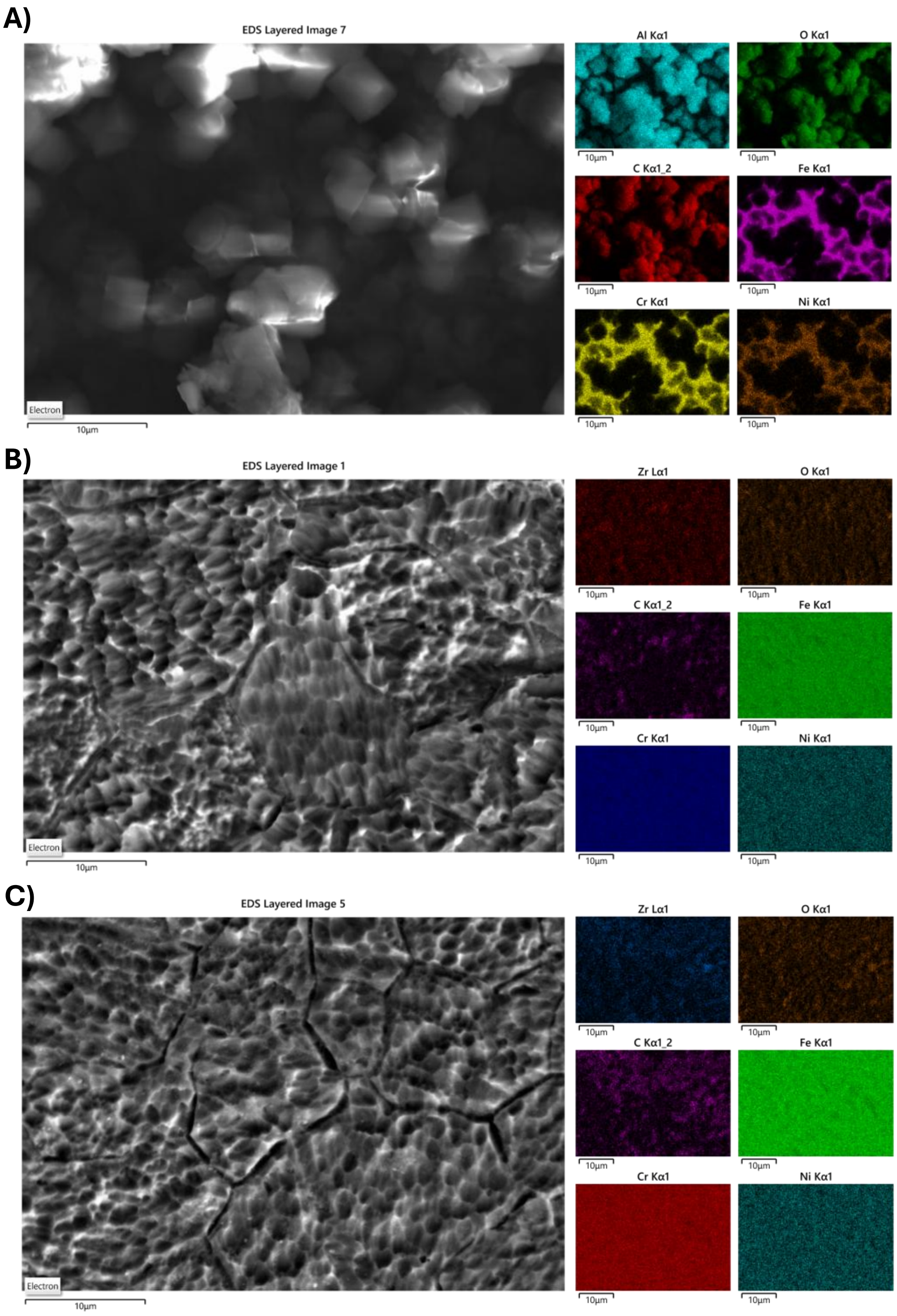

Figure S5. SEM studies of the regions at the edges of the MOF-coated blades, with clear observation of the formation of a preliminary crystal bed.

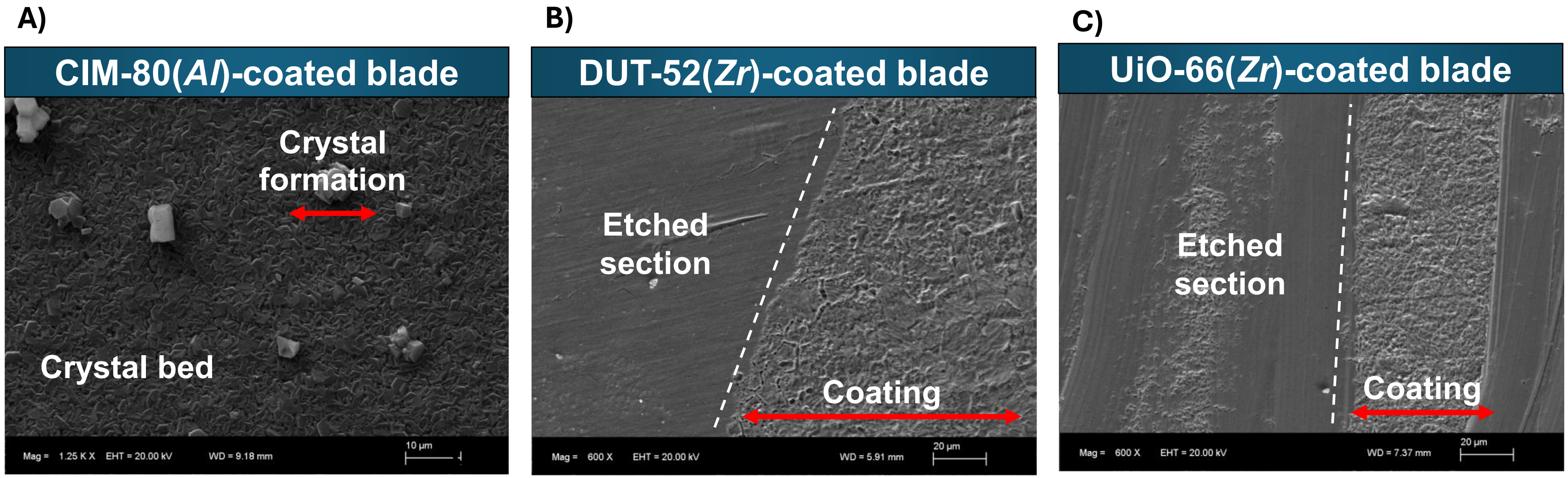

Figure S6. Stability of MOFs when exposed to different complex samples and pH values, evaluated by PXRD studies. The stability tests for the MOFs in different matrices and media were carried out following the protocol described by Pacheco-Fernández *et al.* [Ref. 22 of the main manuscript] with slight variations. Briefly, 40 mg of the three different MOF are immersed in a 20 mL falcon tube containing different aqueous solutions at different pH values (1, 4 and 8), or real biological samples such as saliva (dilution 1:9), plasma (dilution 1:9), and urine (dilution 1:4). MOFs remain in contact with the matrix for 24 h with shaker agitation (500 min^{-1}). In the case of DUT-52(*Zr*), experiments after immersion of the MOF during 3 min were obtained, to mimic the procedure followed in this work. Then, the MOFs powders are cleaned in triplicate with: 1) the solvent used for the MOF's synthesis, 2) water (to remove possible salts from biological matrixes), and 3) ethanol. Lastly, MOFs are dried in an oven at 70 °C before the PXRD characterization. (**A**) CIM-80(*Al*), (**B**) UiO-66(*Zr*) and (**C**) DUT-52(*Zr*).

(**A**)

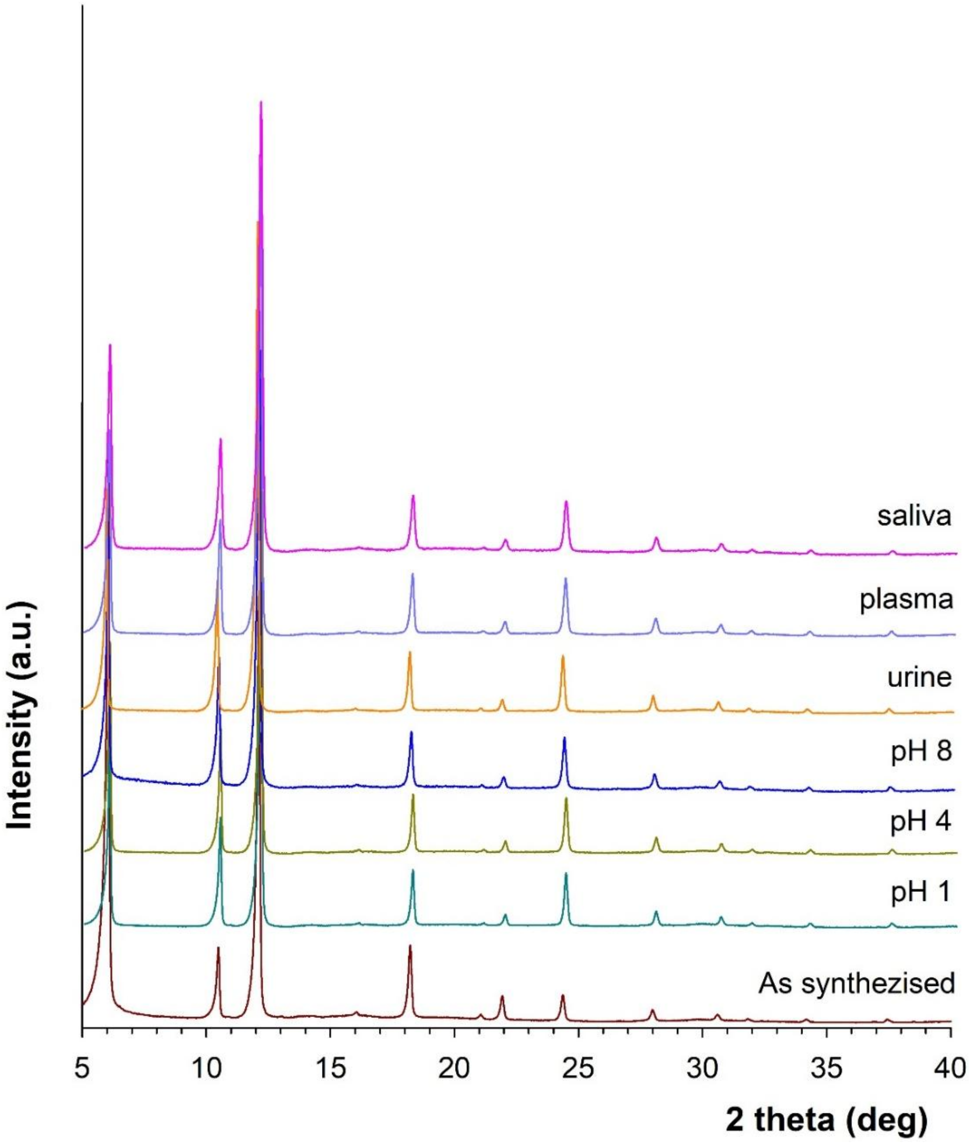

(**B**) DUT-52(*Zr*)

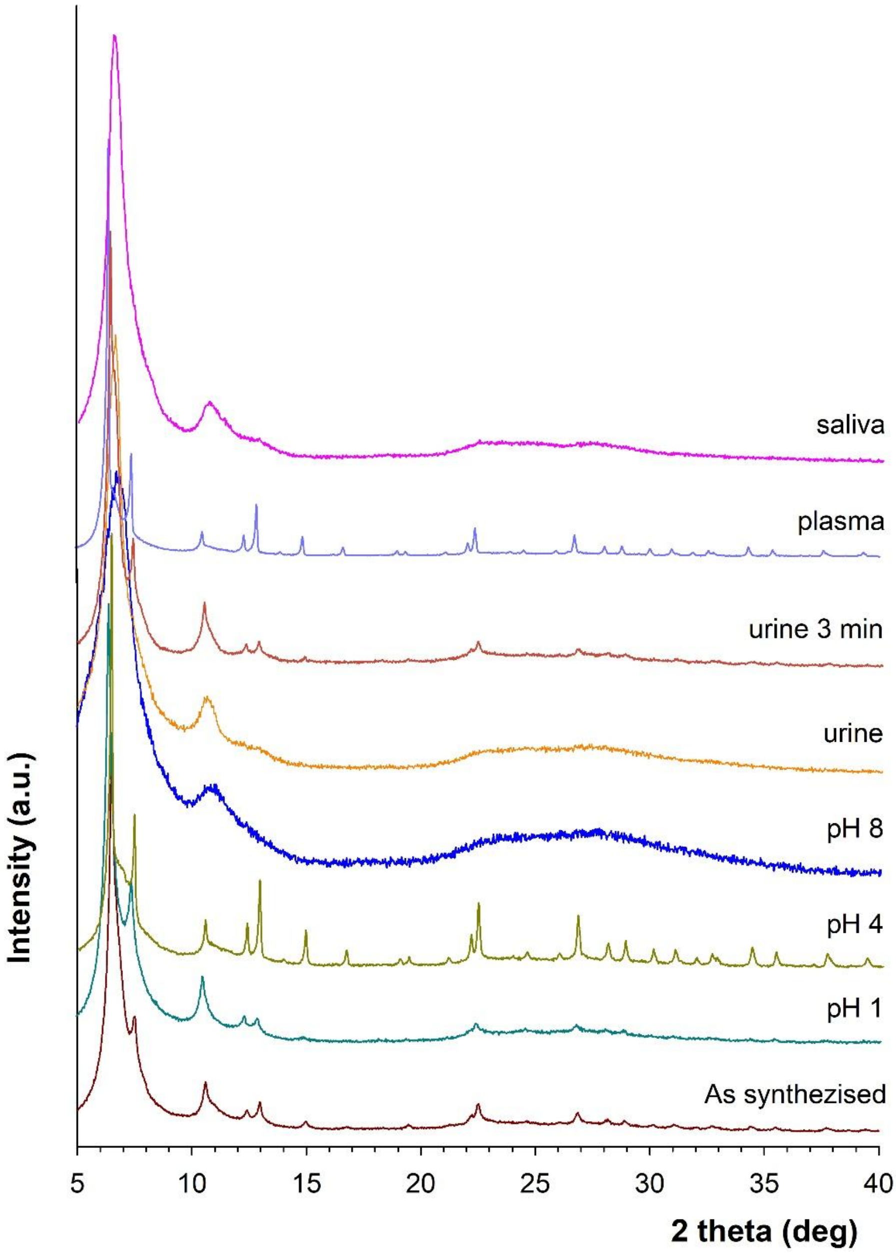
saliva
plasma
urine 3 min
urine
pH 8
pH 4
pH 1
As synthezised
Intensity (a.u.)
5
10
15
20
25
30
35
40
2 theta (deg)

(**C**) UiO-66(*Zr*)

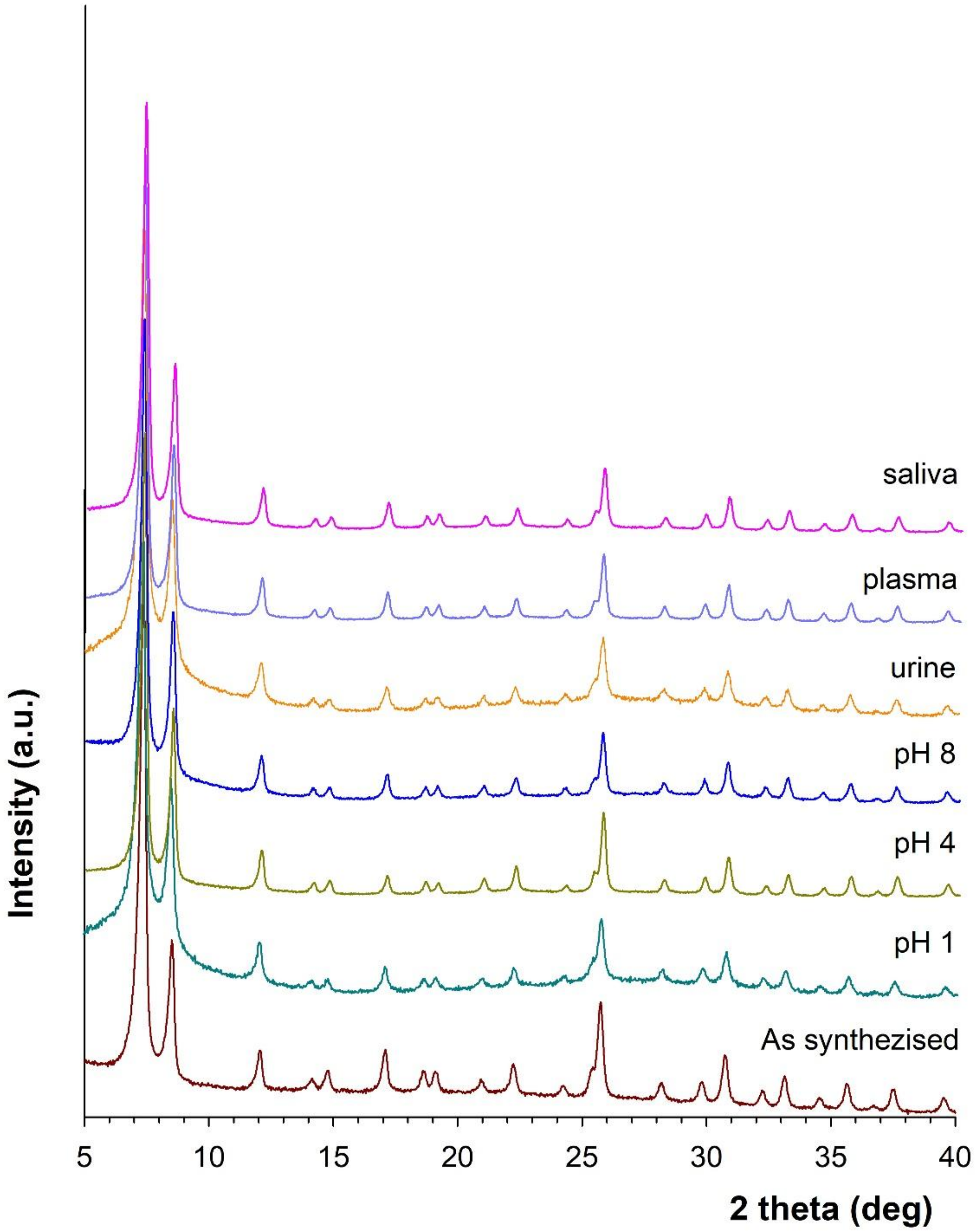
saliva
plasma
urine
pH 8
pH 4
pH 1
As synthezised
Intensity (a.u.)
5
10
15
20
25
30
35
40
2 theta (deg)

Figure S7. Influence of voltage and nature of the desorption solvent on the efficiency obtained, being **A)** IPA and **B)** MeOH.

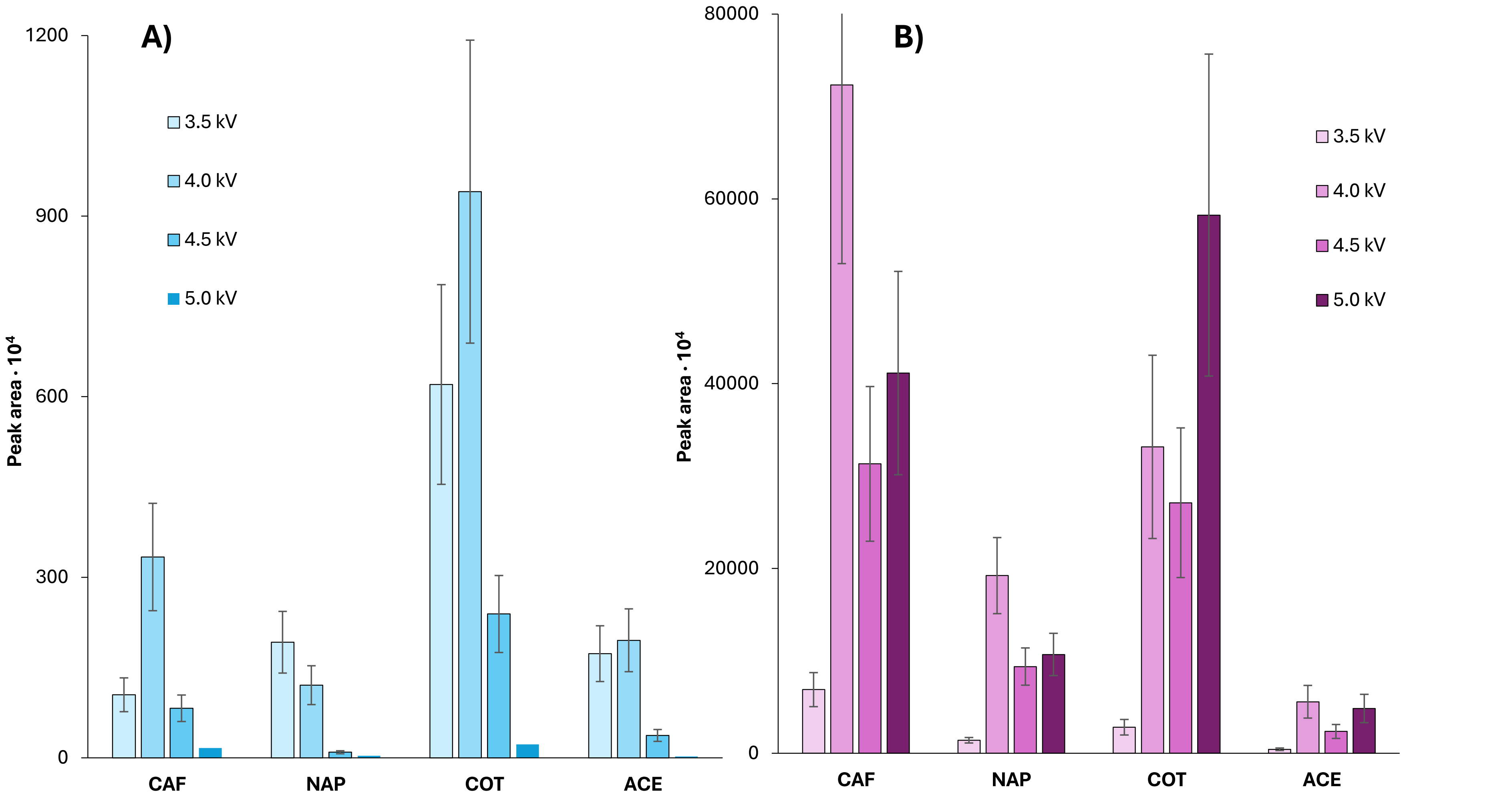

Figure S8. Desorption solvent comparison between MeOH and MeOH:IPA (1:1, *v/v*).

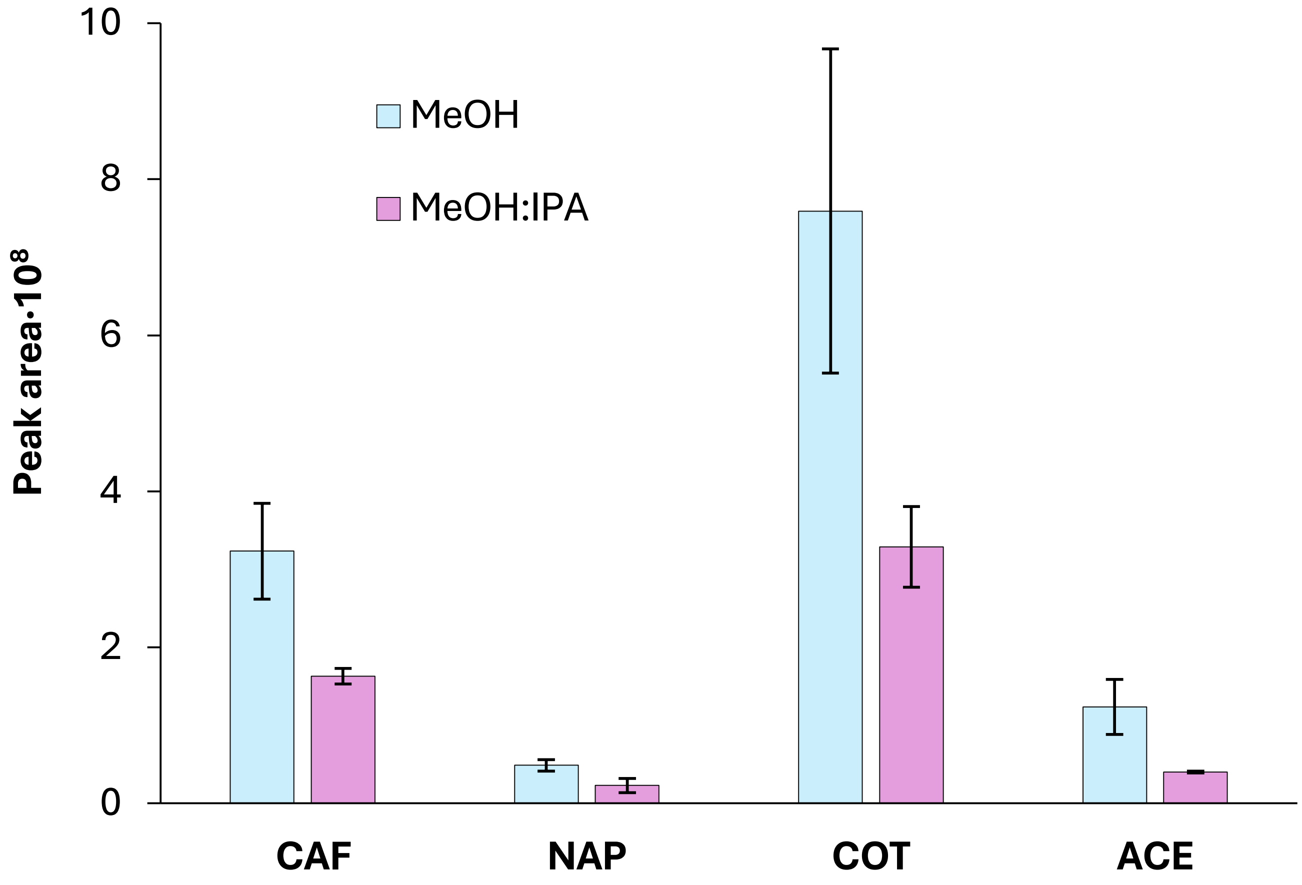

Figure S9. MOF-coated blades device images and contact angle studies.

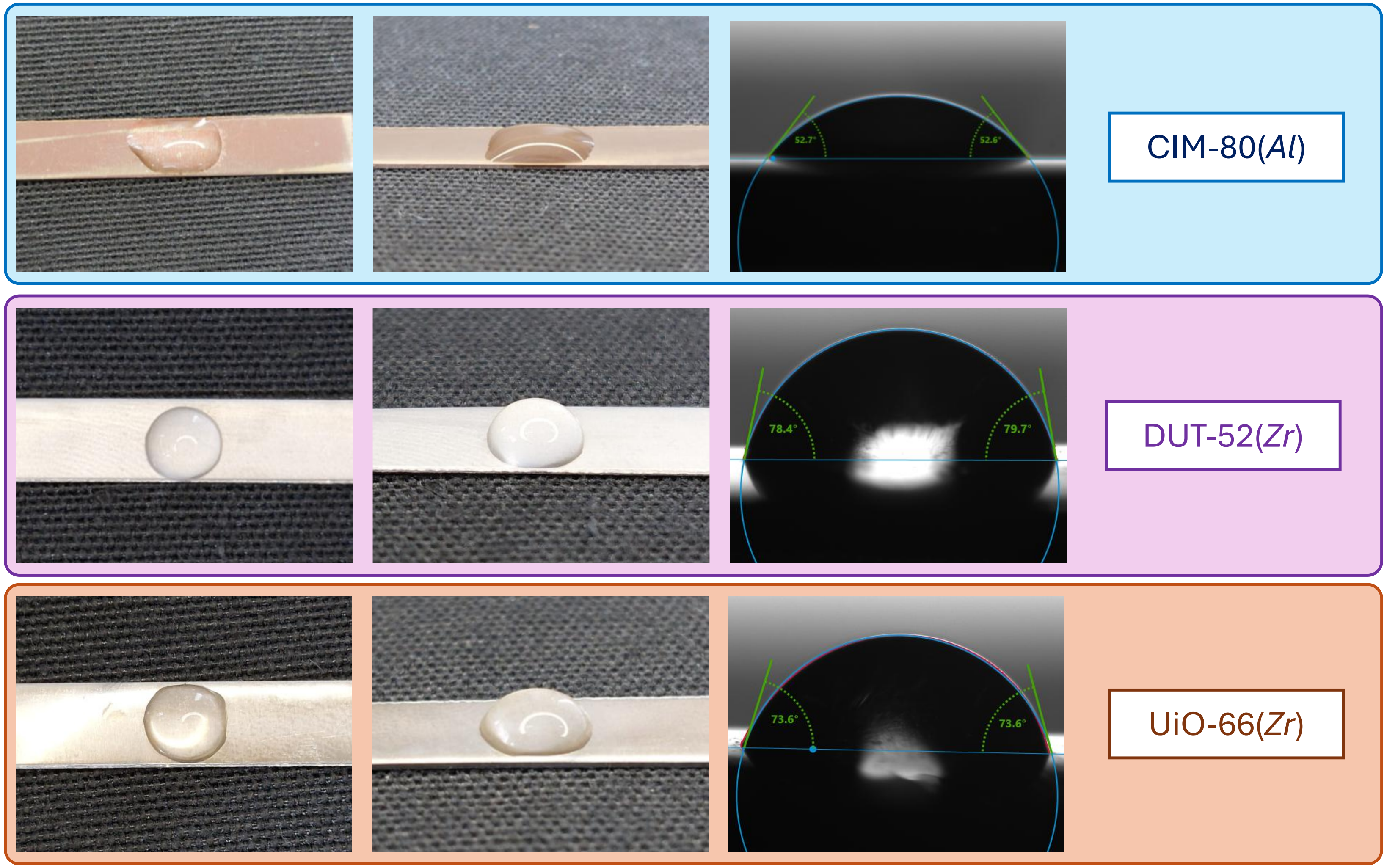

Figure S10. Score metrics of the CBS(MOF)-HRMS of **A**) sustainability and **B**) practicality.

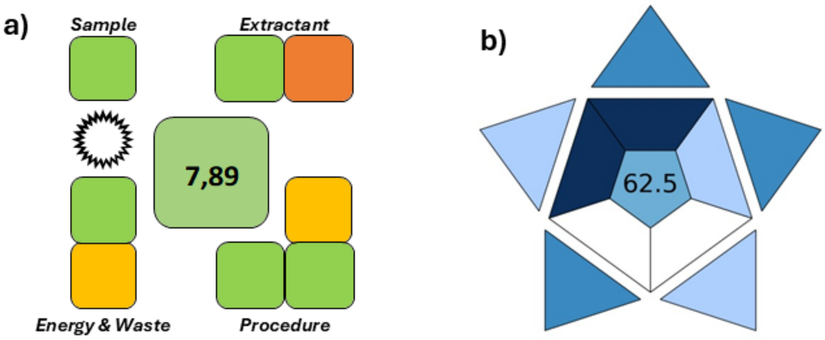

SPMS conditions	
Sample amount	
Sample amount (mL or g)	≤ 10
Extractant information	
Amount of extractant (mL or g)	≤ 0.1
Nature of extractant	Alternative persistant
Procedure information	
Number of steps	2 < x ≤ 4
Extraction time (min)	≤ 5
Additional steps after extraction	No additional steps
Sample throughput	Single sample
Energy consumption	
Dispersion/stir	Shaker
Separation	No centrifuge
Temperature	Room temperature
Total waste	
Waste (mL or g)	≤ 10
Reusability of extractant	
Reusable	Yes

Global score
7.89

BAGI conditions
Type of analysis
Quantitative and confirmatory
Multi- or single-element analysis
Multi-element analysisis for >15 compounds
Analytical technique
Instrumentation that is not commonly available in most labs (SFC, 2D-GC, 2D-LC, LC-MS/MS, GC-MS/MS…)
Simultaneous sample preparation
1
Sample preparation
Miniaturized extraction sample preparation (SPME, DLLME, MEPS, SBSE, d-SPE, FPSE, etc)
Samples per h
5-10
Reagents and materials
Need to be synthesized in the lab with common instrumentation and in a simple way
Preconcentration
Preconcentration required. Required sensitivity is met with one-step preconcentration
Degree of automation
Semi-automated with non-common devices (e.g. homemade systems)
Amount of sample
100-500 µL (or mg) bioanalytical simples; >100 mL (or g) food/environmental

Global score
62.5

Figure S11. Voltage-pulsed acquisition to increase both repeatability and automatization of the CBS(MOF)-HRMS approach.

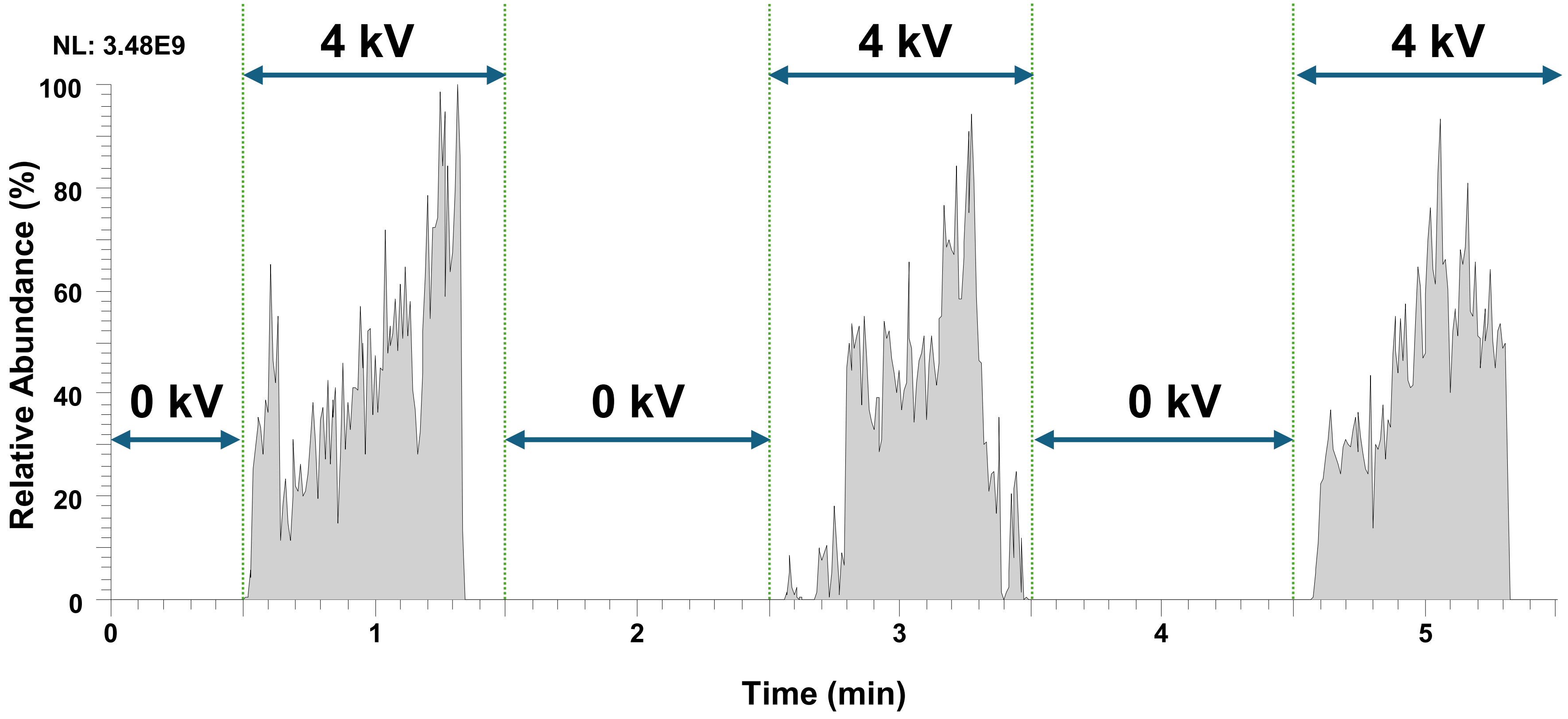

Figure S12. MS/HRMS spectra of the exogenous/endogenous pairs cotinine and serotonin.

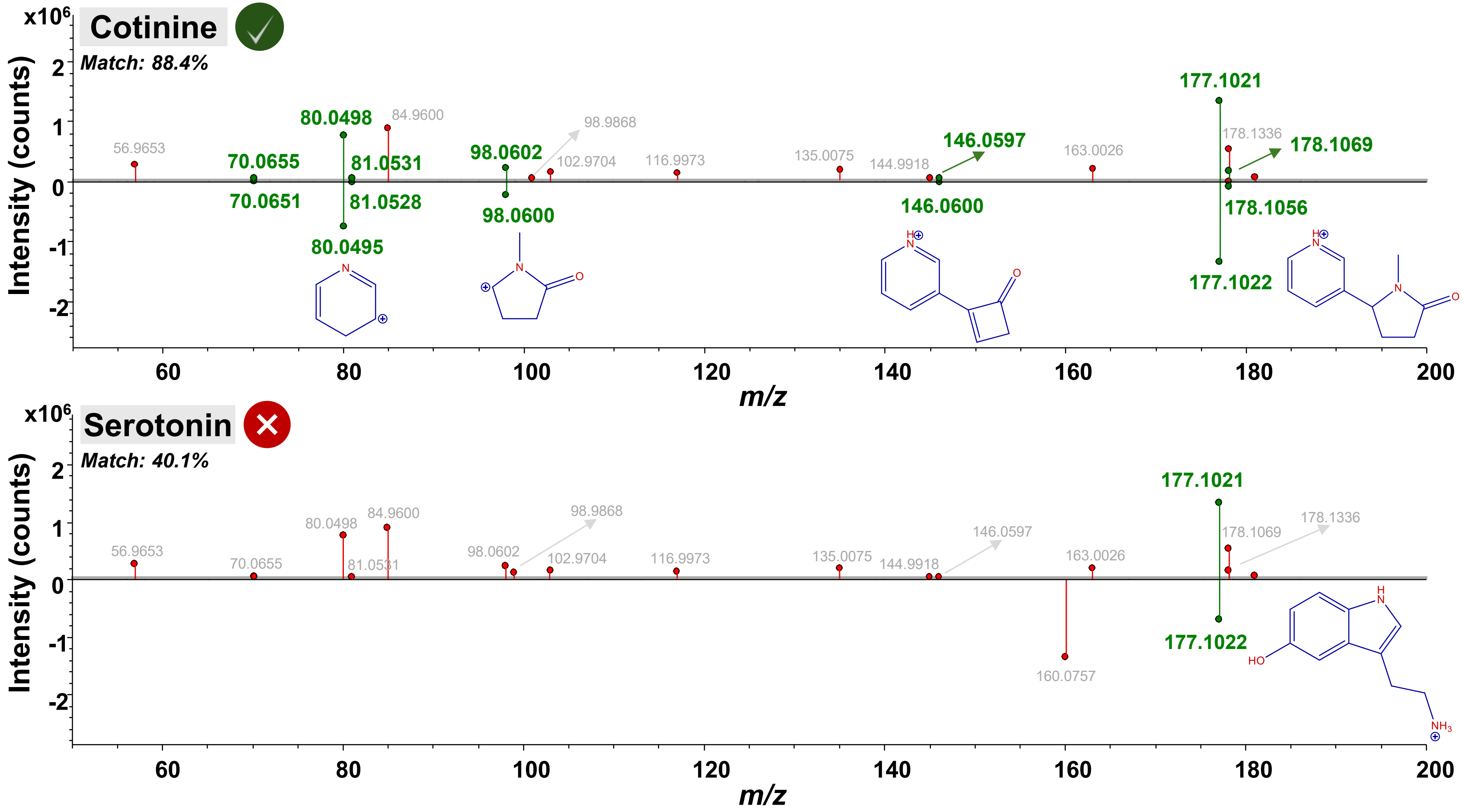

Table S1. Synthetic conditions to prepare the MOF-coatings for the blades.

MOF	Metallic salt / mg	Organic ligand / mg	Modulator / amount	Solvent / mL	Temp. / Time
CIM-80(*Al*)	$Al(NO_3)_3·9H_2O$ / 375	mesaconic acid / 130	urea / 30 mg	water / 15	150 ºC / 3 h
DUT-52(*Zr*)	$ZrCl_4$ / 230	2,6-naphtalene-dicarboxylic acid / 216	acetic acid / 3 mL	DMF / 20	120 °C / 24 h
UiO-66(*Zr*)	$ZrCl_4$ / 125	terephthalic acid / 123	HCl / 1 mL	DMF / 15	120 °C / 24 h

Table S2. Elemental composition of MOF-coated blades determined by SEM-EDX.

MOF-coated blade	Element	% Weight	% Atomic
**CIM-80(*Al*)**	Al	11.54	8.29
	O	23.30	28.23
	C	32.16	51.89
	Cr	7.05	2.63
	Fe	23.60	8.19
	Ni	2.35	0.78
**DUT-52(*Zr*)**	Zr	0.29	0.14
	O	1.03	2.77
	C	7.11	25.55
	Fe	66.12	51.10
	Cr	18.23	15.13
	Ni	7.23	5.32
**UiO-66(*Zr*)**	Zr	0.70	0.33
	O	1.52	4.10
	C	6.75	24.30
	Fe	65.95	51.06
	Cr	18.12	15.07
	Ni	6.97	5.13

Table S3. Inter-device reproducibility obtained with three different DUT-52(Zr)-based blades spiked at 500 $\mu g{\cdot}L^{-1}$ of the target analytes (together with the internal standards).

Analyte	Ratio total peak area[a] / total peak area[a] of the internal standard			RSD (%)
	Blade 1	Blade 2	Blade 3	
CAF	2.15	2.74	1.86	19.9
NAP	0.79	0.88	0.65	15.1
COT	2.72	2.48	2.97	12.7
ACE	0.41	0.56	0.67	24.0

[a]Sum of the peak areas obtained after the three consecutive desorption steps.

Table S4. Main features of methods involving CBS-HRMS (literature search since 2022).

Coating	Analyte(s) / Sample(s)	Volume Required	Preconc. Time	Desorption Solvent / Volume	Desorption Voltage	Measured Time	Accuracy (%)	LOQ ($ng·mL^{-1}$)	Ref.
HLB[a]	VOCs[b] / honey	4 mL (1 g of honey)	10 min	95:5 MeO:H_2O (v/v) + 12 mM ammonium acetate / 10 µL	4 kV	10 s	-	-	31
GO[c]-SA[d]-Biotin-Ab GO[c]-SA[d] -Biotin-IgG	fluro-quinolone drugs / milk	1 mL (100 µL of milk)	10 min	MeOH + 5% FA[e] / 20 µL	3.6 kV	<1 min	81.9 – 102.1	-	34
HLB[a]-PAN[f]	fentanyl & analogues / human plasma & urine	300 µL of plasma	15 – 20 min	95:5 MeOH/H_2O (v/v) + 0.1 % FA[e] / 15 µL	4.5 – 5 kV	15 s	-	0.05 – 1	14
HLB[a]-PAN[f]	drugs of abuse / saliva	1.5 mL of saliva	10 min	95:5 MeOH/H_2O (v/v) + 0.1 % FA[e] / 8 µL	5.0 kV / -3.8 kV	12 s	72 – 126	0.005 – 10	19

Table S4 (*continued*). Main features of methods involving CBS-HRMS (literature search since 2022).

Coating	Analyte(s) / Sample(s)	Volume Required	Preconc. Time	Desorption Solvent / Volume	Desorption Voltage	Measured Time	Accuracy (%)	LOQ ($ng \cdot mL^{-1}$)	Ref.
Cu-TCPP[g]/ $Ti_3C_2T_x$	drugs of abuse / environm. water	1.5 mL of environmental water	20 min	95:5 MeOH/H_2O (v/v) + 0.1 % FA[e] / 8 µL	5 kV	18 s	67.6 – 133.2	0.005 – 0.030	25
HLB[a]-PAN[f]	drugs / urine	*1.5 mL* (0.75 mL of urine)	10 min	95:5 MeOH/H_2O (v/v) / 8 µL	5 kV	35 s	85 – 113	0.1 – 10	20
HLB[a]-PAN[f]	drugs / blood	20 µL of blood	10 min	95:5 MeOH/H_2O (v/v) + 0.1 % FA[e]	5 kV	30 s	-	0.25 – 25	33
HLB[a]	drugs of abuse, pesticides / urine	300 µL of urine	15 min	95:5 MeOH/H_2O (v/v) + 0.1 % FA[e] / 5 µL	5.5 kV	10 s	94 – 120	0.5 – 10	2

Table S4 (*continued*). Main features of methods involving CBS-HRMS (literature search since 2022).

Coating	Analyte(s) / Sample(s)	Volume Required	Preconc. Time	Desorption Solvent / Volume	Desorption Voltage	Measured Time	Accuracy (%)	LOQ ($ng \cdot mL^{-1}$)	Ref.
CIM-80(*Al*)<br>DUT-52(*Zr*)<br>UiO-66(*Zr*)	pharma. drugs & industrial compounds / urine	1.5 mL (300 μL of urine)	3 min	MeOH + 0.1 % FA[e] / 40 – 50 μL	4 kV	1 min	73.3 – 133.25	0.1 – 40	This study

[a]HLB: hydrophilic–lipophilic balance

[b]VOCs: volatile organic compounds

[c]GO: graphene oxide

[d]SA: streptavidin

[e]FA: formic acid

[f]PAN: polyacrylonitrile

[g]TCPP: 4,4′,4′,4′-(porphine-5,10,15,20-tetrayl) tetrakis (benzoic acid)

Table S5. Compounds tentatively identified in urine samples by CBS(MOF-coated blades)-HRMS.

Name	Formula	Error (ppm)	*m/z*	Ion	mzCloud[a] / In-silico Match[b] (%)	Max Abundance	Ident. Level	Compound Class
6-methyl-2-pyridinemethanol/ 5-amino-2-methylphenol	C_7H_9NO	0.1	124.0757	$[M+H]^+$	86.6 / 70.6[c]	U02	3	industrial chemical/personal care product
5-methylcytosine	$C_5H_7N_3O$	-0.7	126.0661	$[M+H]^+$	92.0	U02	2a	endogenous metabolite
creatine	$C_4H_9N_3O_2$	-0.3	132.0767	$[M+H]^+$	83.7	U08	2a	endogenous metabolite
allopurinol/ hypoxanthine	$C_5H_4N_4O$	-1.0	137.0457	$[M+H]^+$	70.0	U02	3	medical drug/ endogenous metabolite
1-methylnicotinamide	$C_7H_8N_2O$	-0.6	137.0709	$[M+H]^+$	99.1	U07	2a	endogenous metabolite
anthranilic acid/ 4-aminobenzoic acid	$C_7H_7NO_2$	-1.1	138.0548	$[M+H]^+$	85.0	U02	3	drugs of abuse/ endogenous metabolite; industrial chemicals
methylimidazoleacetic acid	$C_6H_8N_2O_2$	-1.1	141.0657	$[M+H]^+$	92.5	U02	2a	endogenous metabolite
DL-stachydrine	$C_7H_{13}NO_2$	-1.5	144.1017	$[M+H]^+$	88.3	U01	3	endogenous metabolite
4-guanidinobutyric acid	$C_5H_{11}N_3O_2$	-0.9	146.0923	$[M+H]^+$	82.9	U06	2a	endogenous metabolite
dopamine quinone/acetaminophen	$C_8H_9NO_2$	-2.1	152.0703	$[M+H]^+$	80.0	U06	3	endogenous metabolite/ medical drug
L-histidine	$C_6H_9N_3O_2$	-0.9	156.0766	$[M+H]^+$	98.4	U02	2a	endogenous metabolite
methylguanine	$C_6H_7N_5O$	-0.7	166.0722	$[M+H]^+$	80.2	U07	3	endogenous metabolite
DL-phenylalanine	$C_9H_{11}NO_2$	-0.9	166.0861	$[M+H]^+$	90.0	U05	3	endogenous metabolite
methylxanthine	$C_6H_6N_4O_2$	-1.6	167.0561	$[M+H]^+$	80.0	U02	3	endogenous metabolite
uric acid	$C_5H_4N_4O_3$	-1.1	169.0354	$[M+H]^+$	82.1	U02	2a	endogenous metabolite
3-methylhistidine	$C_7H_{11}N_3O_2$	-0.9	170.0923	$[M+H]^+$	83.2	U02	2a	endogenous metabolite
pyroquilon	$C_{11}H_{11}NO$	-0.7	174.0912	$[M+H]^+$	75.0	U02	2b	pesticide; herbicide

Table S5 (*continued*). Compounds tentatively identified in urine samples by CBS(MOF-coated blades)-HRMS.

**Name**	**Formula**	**Error (ppm)**	***m/z***	**Ion**	**mzCloud[a] / In-silico Match[b] (%)**	**Max Abundance**	**Ident. Level**	**Compound Class**
DL-arginine	$C_6H_{14}N_4O_2$	-0.8	175.1188	$[M+H]^+$	81.0	U06	2a	endogenous metabolite
cotinine	$C_{10}H_{12}N_2O$	-0.9	177.1021	$[M+H]^+$	88.4	U04	1	drug metabolite
paraxanthine/theophylline	$C_7H_8N_4O_2$	-0.8	181.0719	$[M+H]^+$	95.1/94.5[c]	U02	3	medical drug and drug metabolite
caffeine	$C_8H_{10}N_4O_2$	-0.6	195.0875	$[M+H]^+$	88.5	U07	1	industrial chemical
dodecanamide	$C_{12}H_{25}NO$	-1.2	200.2006	$[M+H]^+$	100	U02	2b	food consumption biomarker
$C_{11}H_{23}NO_2$		-0.7	202.1800	$[M+H]^+$	70.0	U07	3	endogenous metabolite
N3,N4-dimethyl-L-arginine	$C_8H_{18}N_4O_2$	-0.9	203.1501	$[M+H]^+$	80.2	U07	2a	endogenous metabolite
naproxen	$C_{14}H_{14}O_3$	2.3	231.1021	$[M+H]^+$	80.3	U09	1	medical drug
N6-methyladenosine	$C_{11}H_{15}N_5O_4$	-1.1	282.1194	$[M+H]^+$	86.7	U07	2a	endogenous metabolite
oleamide	$C_{18}H_{35}NO$	-2.2	282.2785	$[M+H]^+$	98.6	U02	2a	endogenous metabolite
cetrimonium	$C_{19}H_{41}N$	-2.0	284.3306	$[M+H]^+$	83.3	U07	2a	personal care product
palmitoyl ethanolamide/sphingosine	$C_{18}H_{37}NO_2$	-2.4	300.2890	$[M+H]^+$	90.7 / 76.3[c]	U05	3	medical drug/endogenous metabolite
methyl ricinoleate/glycidyl palmitate	$C_{19}H_{36}O_3$	-2.0	313.2731	$[M+H]^+$	100	U01	3	fragance/endogenous metabolite
17-propyl-5α-androst-2-en-17β-ol	$C_{22}H_{36}O$	-2.2	317.2832	$[M+H]^+$	70.0	U06	2b	steroid
dalcotidine	$C_{18}H_{29}N_3O_2$	3.6	320.2344	$[M+H]^+$	70.0	U07	2b	medical drug
stearoyl ethanolamide	$C_{20}H_{41}NO_2$	-2.5	328.3202	$[M+H]^+$	86.9	U05	2a	endogenous metabolites
lauryl glucoside	$C_{18}H_{36}O_6$	-2.2	349.2577	$[M+H]^+$	100	U01	2b	personal care product
lauryl oleate	$C_{30}H_{58}O_2$	-1.5	451.4503	$[M+H]^+$	100	U08	2b	personal care product
14,16-hentriacontanedione	$C_{31}H_{60}O_2$	-1.9	465.4657	$[M+H]^+$	80.0	U06	2b	food consumption biomarker

Table S5 (*continued*). Compounds tentatively identified in urine samples by CBS(MOF-coated blades)-HRMS.

Name	Formula	Error (ppm)	*m/z*	Ion	mzCloud[a] / In-silico Match[b] (%)	Max Abundance	Ident. Level	Compound Class
16,18-tritriacontanedione	$C_{33}H_{64}O_2$	-1.7	493.4971	$[M+H]^+$	90.0	U06	2b	industrial chemical
hexadecyl octadec-9-enoate	$C_{34}H_{66}O_2$	-0.6	507.5132	$[M+H]^+$	100	U07	2b	personal care product
oleyl oleate	$C_{36}H_{68}O_2$	-1.5	533.5284	$[M+H]^+$	90.0	U06	2b	personal care product
oleyl stearate	$C_{36}H_{70}O_2$	-1.5	535.5441	$[M+H]^+$	100	U06	2b	personal care product
ceramide (d:18:1/16:0)/ N-[(4E)-1,3-dihydroxy-4-octadecen-2-yl] hexadecanamid	$C_{34}H_{67}NO_3$	-1.6	538.5185	$[M+H]^+$	100	U05	3	endogenous metabolite
bemotrizinol	$C_{38}H_{49}N_3O_5$	-2.6	628.3729	$[M+H]^+$	80.0	U01	2b	UV filter

[a]For annotations with level 1 and 2a

[b]For annotations with level 2b and 3

[c]mzCloud matches for isomeric compounds
